# Checklist of the family Zopheridae (Coleoptera, Tenebrionoidea) of Bulgaria

**DOI:** 10.3897/BDJ.14.e192174

**Published:** 2026-04-28

**Authors:** Denis Gradinarov, Yana Petrova, Ognyan Sivilov, Vladimir Stefanovich Stefanov

**Affiliations:** 1 Sofia University “St Kliment Ohridski”, Sofia, Bulgaria Sofia University “St Kliment Ohridski” Sofia Bulgaria; 2 National Genetic Laboratory, Sofia, Bulgaria National Genetic Laboratory Sofia Bulgaria; 3 Faculty of Forestry, University of Forestry, Sofia, Bulgaria Faculty of Forestry, University of Forestry Sofia Bulgaria

**Keywords:** Tenebrionoidea, Zopheridae, distribution, Bulgaria

## Abstract

**Background:**

Beetles of the family Zopheridae (Coleoptera, Tenebrionoidea) are rarely reported from Bulgaria and available data on their distribution in the country are scarce. Here, we provide an updated checklist of the family, based on published data and new records.

**New information:**

Twenty-one species of the family Zopheridae are listed for Bulgaria. New data on the distribution of fourteen species are provided. The species *Colydium noblecourti* Parmain, Eckelt & Schuh, 2024 is reported for the first time for the country. The presence of the species *Aulonium trisulcum* (Geoffroy, 1785), *A. ruficorne* (G.-A. Olivier, 1790), *Orthocerus clavicornis* (Linnaeus, 1758), *Langelandia anophthalma* Aubé, 1842 and *Synchita mediolanensis* A. Villa & J. B. Villa, 1833 is confirmed for Bulgaria.

## Introduction

According to the second edition of the Catalogue of Palaearctic Coleoptera, 16 species of the family Zopheridae (Coleoptera, Tenebrionoidea) are known for Bulgaria (Schuh 2020). Нowever, some of the species records of earlier authors are omitted in the Catalogue and, for other species, there are no available published localities from Bulgaria (Guéorguiev et al. 2026). Species of Zopheridae are rarely reported from Bulgaria and special taxonomic or faunistic studies of the family in the country have not been yet conducted.

In the present work, we summarise all available literature records of the species of Zopheridae in Bulgaria and provide new distributional data for fourteen species. Original photographs of all examined species are provided. The prepared checklist also contains updated data on the global distribution of each species.

## Materials and methods

The main part of the material for this study was collected by the authors in the period 1999 – 2025 from different regions of Bulgaria. Materials of other collectors, preserved in the Zoological Collection of Sofia University “St Kliment Ohridski”, Faculty of Biology, Sofia, Bulgaria (BFUS), are also examined. The newly-collected specimens are catalogued and deposited in the BFUS collection.

The identification of the newly-collected material was carried out by external morphology characteristics and, in cases where this was necessary, by the morphology of the male genitalia. The identification keys available in Kryzhanovsky (1965); Ivie et al. (2016); Lompe (2026) were used, as well as revisions and taxonomic studies of the genera and species, as follows: Węgrzynowicz (1999); Parmain et al. (2024) (for the genus *Colydium* Fabricius, 1792); Dajoz (1971); Jelínek (1981); Soldati et al. (2018) (for the genus *Bitoma* Herbst, 1793); Reitter (1912); Dajoz (1968); Dajoz (1969); Orousset (1989) (for the genus *Langelandia* Aubé, 1842); Schuh (1998); Ruta (2020) (for the genus *Synchita* Hellwig, 1792).

The historical collection of Dimitar Joakimov, preserved in the Entomological collection of the National Museum of Natural History, Sofia, Bulgaria (NMNHS) was examined to clarify the species composition of the genus *Colydium* Fabricius, 1792 in Bulgaria.

The photographs of the beetles were taken with a Canon EOS R50 camera with Laowa 25 mm f/2.8 2.5-5X Ultra Macro or Laowa 90 mm f/2.8 2x Ultra Macro APO lens, mounted on a WeMacro rail (Wemacro, Shanghai, China). The photographs of genital structures were taken with a Canon EOS R50 camera with Laowa Aurogon FF 10-50X NA0.5 Supermicro APO lens or with Leica K3C camera attached to Leica DM750 microscope.

## Checklists

### Checklist of the Zopheridae of Bulgaria

#### Coleoptera

Linnaeus, 1758

88E0023A-1CC7-5933-BD36-CD1F07E67541

#### Tenebrionoidea

Latreille, 1802

959F88C3-BF80-559D-9D1E-1105807EE3FF

#### Zopheridae

Solier, 1834

75098206-4A18-50AB-8EBD-ED6B97523824

#### Zopherinae

Solier, 1834

469098FC-BF17-5765-9AD4-233CD6D06891

#### Pycnomerini

Erichson, 1845

CFC9105A-3732-5C83-98E5-1125EA38D038

#### Pycnomerus

Erichson, 1842

1C4F4943-C957-56B3-84BF-A078261F43D7

#### Pycnomerus sulcicollis

(Germar, 1823)

794260A7-D77D-5ADA-8BF6-BC0CCA2DCA34

##### Materials

- **Type status:** Other material. **Occurrence:** catalogNumber: BFUS-COL000728; recordedBy: Plamen Mitov, Rumyana Kostova & Ognyan Sivilov; individualCount: 1; sex: male; occurrenceID: EB39599A-F863-5DA2-AAA6-94A37D84D018; **Location:** country: Bulgaria; stateProvince: Burgas; municipality: Malko Tarnovo; locality: Strandzha Mountains, SW of Slivarovo Village; verbatimElevation: 220 m; decimalLatitude: 41.960500; decimalLongitude: 27.659667; geodeticDatum: WGS84; **Identification:** identifiedBy: Denis Gradinarov; dateIdentified: 2026; **Event:** samplingProtocol: pitfall traps; verbatimEventDate: 15.iv.2009–08.v.2009; habitat: riverine forest
- **Type status:** Other material. **Occurrence:** catalogNumber: BFUS-COL000729; recordedBy: Plamen Mitov, Rumyana Kostova & Ognyan Sivilov; individualCount: 1; sex: specimen; occurrenceID: 40551079-9583-5E41-B7D3-74084D638B76; **Location:** country: Bulgaria; stateProvince: Burgas; municipality: Malko Tarnovo; locality: Strandzha Mountains, SW of Slivarovo Village; verbatimElevation: 220 m; decimalLatitude: 41.960500; decimalLongitude: 27.659667; geodeticDatum: WGS84; **Identification:** identifiedBy: Denis Gradinarov; dateIdentified: 2026; **Event:** samplingProtocol: pitfall traps; verbatimEventDate: 15.iv.2009–08.v.2009; habitat: riverine forest
- **Type status:** Other material. **Occurrence:** catalogNumber: BFUS-COL000730; recordedBy: Plamen Mitov, Rumyana Kostova & Ognyan Sivilov; individualCount: 1; sex: specimen; occurrenceID: 6C387AF2-D375-5C72-B8FD-F3ED15354219; **Location:** country: Bulgaria; stateProvince: Burgas; municipality: Malko Tarnovo; locality: Strandzha Mountains, SW of Slivarovo Village; verbatimElevation: 220 m; decimalLatitude: 41.960500; decimalLongitude: 27.659667; geodeticDatum: WGS84; **Identification:** identifiedBy: Denis Gradinarov; dateIdentified: 2026; **Event:** samplingProtocol: pitfall traps; verbatimEventDate: 15.iv.2009–08.v.2009; habitat: riverine forest
- **Type status:** Other material. **Occurrence:** catalogNumber: BFUS-COL000731; recordedBy: Plamen Mitov, Rumyana Kostova & Ognyan Sivilov; individualCount: 1; sex: specimen; occurrenceID: ABAD9BF6-6A72-5CB7-B762-22A1C2DEF1D2; **Location:** country: Bulgaria; stateProvince: Burgas; municipality: Malko Tarnovo; locality: Strandzha Mountains, SW of Slivarovo Village; verbatimElevation: 220 m; decimalLatitude: 41.960500; decimalLongitude: 27.659667; geodeticDatum: WGS84; **Identification:** identifiedBy: Denis Gradinarov; dateIdentified: 2026; **Event:** samplingProtocol: pitfall traps; verbatimEventDate: 15.iv.2009–08.v.2009; habitat: riverine forest
- **Type status:** Other material. **Occurrence:** catalogNumber: BFUS-COL000732; recordedBy: Plamen Mitov, Rumyana Kostova & Ognyan Sivilov; individualCount: 1; sex: specimen; occurrenceID: CCA76659-7709-5440-8E31-D6369BAF0437; **Location:** country: Bulgaria; stateProvince: Burgas; municipality: Malko Tarnovo; locality: Strandzha Mountains, SW of Slivarovo Village; verbatimElevation: 220 m; decimalLatitude: 41.960500; decimalLongitude: 27.659667; geodeticDatum: WGS84; **Identification:** identifiedBy: Denis Gradinarov; dateIdentified: 2026; **Event:** samplingProtocol: pitfall traps; verbatimEventDate: 15.iv.2009–08.v.2009; habitat: riverine forest
- **Type status:** Other material. **Occurrence:** catalogNumber: BFUS-COL000733; recordedBy: Plamen Mitov, Rumyana Kostova & Ognyan Sivilov; individualCount: 1; sex: specimen; occurrenceID: C45B4517-5895-5CC5-9083-0A87D6EAE56A; **Location:** country: Bulgaria; stateProvince: Burgas; municipality: Malko Tarnovo; locality: Strandzha Mountains, SW of Slivarovo Village; verbatimElevation: 220 m; decimalLatitude: 41.960500; decimalLongitude: 27.659667; geodeticDatum: WGS84; **Identification:** identifiedBy: Denis Gradinarov; dateIdentified: 2026; **Event:** samplingProtocol: pitfall traps; verbatimEventDate: 15.iv.2009–08.v.2009; habitat: riverine forest
- **Type status:** Other material. **Occurrence:** catalogNumber: BFUS-COL000734; recordedBy: Plamen Mitov, Rumyana Kostova & Ognyan Sivilov; individualCount: 1; sex: female; occurrenceID: CF65BB12-E551-560F-BA0E-BFF8E9640A6B; **Location:** country: Bulgaria; stateProvince: Burgas; municipality: Tsarevo; locality: Strandzha Mountains, NE of Balgari Village; verbatimElevation: 180 m; decimalLatitude: 42.111583; decimalLongitude: 27.764717; geodeticDatum: WGS84; **Identification:** identifiedBy: Denis Gradinarov; dateIdentified: 2026; **Event:** samplingProtocol: pitfall traps; verbatimEventDate: 8.v.2009–08.vi.2009; habitat: beech-oak forest
- **Type status:** Other material. **Occurrence:** catalogNumber: BFUS-COL000735; recordedBy: Ognyan Sivilov; individualCount: 1; sex: male; occurrenceID: E5E273DE-D0C9-51A0-B313-AA9E338ED2AB; **Location:** country: Bulgaria; stateProvince: Blagoevgrad; municipality: Petrich; locality: Belasitsa Mountains, S of Petrich; verbatimElevation: 460 m; decimalLatitude: 41.366581; decimalLongitude: 23.210889; geodeticDatum: WGS84; **Identification:** identifiedBy: Denis Gradinarov; dateIdentified: 2026; **Event:** samplingProtocol: pitfall traps; verbatimEventDate: 06.iv.2012–15.v.2012; habitat: deciduous forest
- **Type status:** Other material. **Occurrence:** catalogNumber: BFUS-COL000736; recordedBy: Ognyan Sivilov; individualCount: 1; sex: female; occurrenceID: D83CC546-DE86-574C-9971-5492595F1732; **Location:** country: Bulgaria; stateProvince: Blagoevgrad; municipality: Petrich; locality: Belasitsa Mountains, S of Petrich; verbatimElevation: 460 m; decimalLatitude: 41.366581; decimalLongitude: 23.210889; geodeticDatum: WGS84; **Identification:** identifiedBy: Denis Gradinarov; dateIdentified: 2026; **Event:** samplingProtocol: pitfall traps; verbatimEventDate: 15.v.2012–16.vi.2012; habitat: deciduous forest
- **Type status:** Other material. **Occurrence:** catalogNumber: BFUS-COL000737; recordedBy: Ognyan Sivilov; individualCount: 1; sex: specimen; occurrenceID: 9FA65618-9B85-58AE-8237-4063C5A44552; **Location:** country: Bulgaria; stateProvince: Blagoevgrad; municipality: Petrich; locality: Belasitsa Mountains, S of Petrich; verbatimElevation: 460 m; decimalLatitude: 41.366581; decimalLongitude: 23.210889; geodeticDatum: WGS84; **Identification:** identifiedBy: Denis Gradinarov; dateIdentified: 2026; **Event:** samplingProtocol: pitfall traps; verbatimEventDate: 16.vi.2012–18.vii.2012; habitat: deciduous forest
- **Type status:** Other material. **Occurrence:** catalogNumber: BFUS-COL000738; recordedBy: Yana Petrova; individualCount: 1; sex: female; occurrenceID: 8C644680-0758-5116-88C5-04285FE2D036; **Location:** country: Bulgaria; stateProvince: Stara Zagora; municipality: Bratya Daskalovi; locality: Sarnena Gora Mountains, NW of Gorno Novo Selo Village; verbatimElevation: 800 m; decimalLatitude: 42.473667; decimalLongitude: 25.221667; geodeticDatum: WGS84; **Identification:** identifiedBy: Denis Gradinarov; dateIdentified: 2020; **Event:** samplingProtocol: under bark; verbatimEventDate: 21.x.2018; habitat: oak forest
- **Type status:** Other material. **Occurrence:** catalogNumber: BFUS-COL000739; recordedBy: Yana Petrova; individualCount: 1; sex: female; occurrenceID: A04FF6C0-A8F4-57DB-AB9A-29188D19446E; **Location:** country: Bulgaria; stateProvince: Stara Zagora; municipality: Bratya Daskalovi; locality: Sarnena Gora Mountains, NW of Gorno Novo Selo Village; verbatimElevation: 800 m; decimalLatitude: 42.473667; decimalLongitude: 25.221667; geodeticDatum: WGS84; **Identification:** identifiedBy: Denis Gradinarov; dateIdentified: 2020; **Event:** samplingProtocol: under bark; verbatimEventDate: 21.x.2018; habitat: oak forest
- **Type status:** Other material. **Occurrence:** catalogNumber: BFUS-COL000740; recordedBy: Yana Petrova; individualCount: 1; sex: specimen; occurrenceID: 98EA0A47-989F-58E4-87D4-940EABBB90E4; **Location:** country: Bulgaria; stateProvince: Stara Zagora; municipality: Bratya Daskalovi; locality: Sarnena Gora Mountains, NW of Gorno Novo Selo Village; verbatimElevation: 800 m; decimalLatitude: 42.473667; decimalLongitude: 25.221667; geodeticDatum: WGS84; **Identification:** identifiedBy: Denis Gradinarov; dateIdentified: 2020; **Event:** samplingProtocol: under bark; verbatimEventDate: 21.x.2018; habitat: oak forest
- **Type status:** Other material. **Occurrence:** catalogNumber: BFUS-COL000741; recordedBy: Yana Petrova; individualCount: 1; sex: specimen; occurrenceID: 40B2A479-072E-5A50-AB72-8BF047BCBCD8; **Location:** country: Bulgaria; stateProvince: Stara Zagora; municipality: Bratya Daskalovi; locality: Sarnena Gora Mountains, NW of Gorno Novo Selo Village; verbatimElevation: 800 m; decimalLatitude: 42.473667; decimalLongitude: 25.221667; geodeticDatum: WGS84; **Identification:** identifiedBy: Denis Gradinarov; dateIdentified: 2020; **Event:** samplingProtocol: under bark; verbatimEventDate: 21.x.2018; habitat: oak forest
- **Type status:** Other material. **Occurrence:** catalogNumber: BFUS-COL000742; recordedBy: Yana Petrova; individualCount: 1; sex: specimen; occurrenceID: B27D5538-AC7A-576A-8E36-DC95D2F45CA3; **Location:** country: Bulgaria; stateProvince: Stara Zagora; municipality: Bratya Daskalovi; locality: Sarnena Gora Mountains, NW of Gorno Novo Selo Village; verbatimElevation: 800 m; decimalLatitude: 42.473667; decimalLongitude: 25.221667; geodeticDatum: WGS84; **Identification:** identifiedBy: Denis Gradinarov; dateIdentified: 2020; **Event:** samplingProtocol: under bark; verbatimEventDate: 21.x.2018; habitat: oak forest
- **Type status:** Other material. **Occurrence:** catalogNumber: BFUS-COL000743; recordedBy: Yana Petrova; individualCount: 1; sex: specimen; occurrenceID: 8E460092-4CBA-53E9-B3E6-F9DF3A867313; **Location:** country: Bulgaria; stateProvince: Stara Zagora; municipality: Bratya Daskalovi; locality: Sarnena Gora Mountains, NW of Gorno Novo Selo Village; verbatimElevation: 800 m; decimalLatitude: 42.473667; decimalLongitude: 25.221667; geodeticDatum: WGS84; **Identification:** identifiedBy: Denis Gradinarov; dateIdentified: 2020; **Event:** samplingProtocol: under bark; verbatimEventDate: 21.x.2018; habitat: oak forest
- **Type status:** Other material. **Occurrence:** catalogNumber: BFUS-COL000744; recordedBy: Yana Petrova; individualCount: 1; sex: specimen; occurrenceID: 21C061FA-0670-5FC1-876A-CE51A3C2DB6B; **Location:** country: Bulgaria; stateProvince: Stara Zagora; municipality: Bratya Daskalovi; locality: Sarnena Gora Mountains, NW of Gorno Novo Selo Village; verbatimElevation: 800 m; decimalLatitude: 42.473667; decimalLongitude: 25.221667; geodeticDatum: WGS84; **Identification:** identifiedBy: Denis Gradinarov; dateIdentified: 2020; **Event:** samplingProtocol: under bark; verbatimEventDate: 21.x.2018; habitat: oak forest
- **Type status:** Other material. **Occurrence:** catalogNumber: BFUS-COL000745; recordedBy: Yana Petrova; individualCount: 1; sex: specimen; occurrenceID: F4D5359E-E11D-5663-845D-C5D6D0DADEE9; **Location:** country: Bulgaria; stateProvince: Stara Zagora; municipality: Bratya Daskalovi; locality: Sarnena Gora Mountains, NW of Gorno Novo Selo Village; verbatimElevation: 800 m; decimalLatitude: 42.473667; decimalLongitude: 25.221667; geodeticDatum: WGS84; **Identification:** identifiedBy: Denis Gradinarov; dateIdentified: 2026; **Event:** samplingProtocol: under bark; verbatimEventDate: 21.x.2018; habitat: oak forest
- **Type status:** Other material. **Occurrence:** catalogNumber: BFUS-COL000746; recordedBy: Yana Petrova; individualCount: 1; sex: specimen; occurrenceID: 1A8D3C6F-93B2-5A90-ACC9-5A3DAEF080C5; **Location:** country: Bulgaria; stateProvince: Stara Zagora; municipality: Bratya Daskalovi; locality: Sarnena Gora Mountains, NW of Gorno Novo Selo Village; verbatimElevation: 800 m; decimalLatitude: 42.473667; decimalLongitude: 25.221667; geodeticDatum: WGS84; **Identification:** identifiedBy: Denis Gradinarov; dateIdentified: 2026; **Event:** samplingProtocol: under bark; verbatimEventDate: 21.x.2018; habitat: oak forest
- **Type status:** Other material. **Occurrence:** catalogNumber: BFUS-COL000747; recordedBy: Yana Petrova; individualCount: 1; sex: specimen; occurrenceID: 5B60E297-2712-50C6-AC23-CC98729E78A8; **Location:** country: Bulgaria; stateProvince: Stara Zagora; municipality: Bratya Daskalovi; locality: Sarnena Gora Mountains, NW of Gorno Novo Selo Village; verbatimElevation: 800 m; decimalLatitude: 42.473667; decimalLongitude: 25.221667; geodeticDatum: WGS84; **Identification:** identifiedBy: Denis Gradinarov; dateIdentified: 2026; **Event:** samplingProtocol: under bark; verbatimEventDate: 21.x.2018; habitat: oak forest
- **Type status:** Other material. **Occurrence:** catalogNumber: BFUS-COL000748; recordedBy: Yana Petrova; individualCount: 1; sex: specimen; occurrenceID: 2ABC0B49-9902-52E8-B2BA-0D7753711519; **Location:** country: Bulgaria; stateProvince: Stara Zagora; municipality: Bratya Daskalovi; locality: Sarnena Gora Mountains, NW of Gorno Novo Selo Village; verbatimElevation: 800 m; decimalLatitude: 42.473667; decimalLongitude: 25.221667; geodeticDatum: WGS84; **Identification:** identifiedBy: Denis Gradinarov; dateIdentified: 2026; **Event:** samplingProtocol: under bark; verbatimEventDate: 21.x.2018; habitat: oak forest
- **Type status:** Other material. **Occurrence:** catalogNumber: BFUS-COL000749; recordedBy: Yana Petrova; individualCount: 1; sex: specimen; occurrenceID: 4C6D23D9-1CC0-5670-B545-9A92243B93FC; **Location:** country: Bulgaria; stateProvince: Stara Zagora; municipality: Bratya Daskalovi; locality: Sarnena Gora Mountains, NW of Gorno Novo Selo Village; verbatimElevation: 800 m; decimalLatitude: 42.473667; decimalLongitude: 25.221667; geodeticDatum: WGS84; **Identification:** identifiedBy: Denis Gradinarov; dateIdentified: 2026; **Event:** samplingProtocol: under bark; verbatimEventDate: 21.x.2018; habitat: oak forest
- **Type status:** Other material. **Occurrence:** catalogNumber: BFUS-COL000750; recordedBy: Yana Petrova; individualCount: 1; sex: specimen; occurrenceID: C9A580C8-0644-53F9-BA66-CCAEE0DD85A0; **Location:** country: Bulgaria; stateProvince: Stara Zagora; municipality: Bratya Daskalovi; locality: Sarnena Gora Mountains, NW of Gorno Novo Selo Village; verbatimElevation: 800 m; decimalLatitude: 42.473667; decimalLongitude: 25.221667; geodeticDatum: WGS84; **Identification:** identifiedBy: Denis Gradinarov; dateIdentified: 2026; **Event:** samplingProtocol: under bark; verbatimEventDate: 21.x.2018; habitat: oak forest
- **Type status:** Other material. **Occurrence:** catalogNumber: BFUS-COL000751; recordedBy: Yana Petrova; individualCount: 1; sex: specimen; occurrenceID: 19C1936B-7A87-5E1B-9F0C-CBE4F960AEF8; **Location:** country: Bulgaria; stateProvince: Stara Zagora; municipality: Bratya Daskalovi; locality: Sarnena Gora Mountains, NW of Gorno Novo Selo Village; verbatimElevation: 800 m; decimalLatitude: 42.473667; decimalLongitude: 25.221667; geodeticDatum: WGS84; **Identification:** identifiedBy: Denis Gradinarov; dateIdentified: 2026; **Event:** samplingProtocol: under bark; verbatimEventDate: 21.x.2018; habitat: oak forest
- **Type status:** Other material. **Occurrence:** catalogNumber: BFUS-COL000752; recordedBy: Yana Petrova; individualCount: 1; sex: specimen; occurrenceID: 9D6636CF-E76B-58D4-AEC8-70AEAD35595F; **Location:** country: Bulgaria; stateProvince: Stara Zagora; municipality: Bratya Daskalovi; locality: Sarnena Gora Mountains, NW of Gorno Novo Selo Village; verbatimElevation: 800 m; decimalLatitude: 42.473667; decimalLongitude: 25.221667; geodeticDatum: WGS84; **Identification:** identifiedBy: Denis Gradinarov; dateIdentified: 2026; **Event:** samplingProtocol: under bark; verbatimEventDate: 21.x.2018; habitat: oak forest
- **Type status:** Other material. **Occurrence:** catalogNumber: BFUS-COL000753; recordedBy: Yana Petrova; individualCount: 1; sex: specimen; occurrenceID: 4565BCB4-A871-50CC-AD88-BA2A85782249; **Location:** country: Bulgaria; stateProvince: Stara Zagora; municipality: Bratya Daskalovi; locality: Sarnena Gora Mountains, NW of Gorno Novo Selo Village; verbatimElevation: 800 m; decimalLatitude: 42.473667; decimalLongitude: 25.221667; geodeticDatum: WGS84; **Identification:** identifiedBy: Denis Gradinarov; dateIdentified: 2026; **Event:** samplingProtocol: under bark; verbatimEventDate: 21.x.2018; habitat: oak forest
- **Type status:** Other material. **Occurrence:** catalogNumber: BFUS-COL000754; recordedBy: Yana Petrova; individualCount: 1; sex: specimen; occurrenceID: F8D5A786-D6E7-5A51-BB8B-9B379C628E56; **Location:** country: Bulgaria; stateProvince: Stara Zagora; municipality: Bratya Daskalovi; locality: Sarnena Gora Mountains, NW of Gorno Novo Selo Village; verbatimElevation: 800 m; decimalLatitude: 42.473667; decimalLongitude: 25.221667; geodeticDatum: WGS84; **Identification:** identifiedBy: Denis Gradinarov; dateIdentified: 2026; **Event:** samplingProtocol: under bark; verbatimEventDate: 21.x.2018; habitat: oak forest

##### Distribution

Azerbaijan, Armenia, Bulgaria, Georgia, Greece, Romania, South European Territory of Russia, Ukraine, Asian Türkiye, Iran (Schuh 2020); Belgium (Thomaes et al. 2025).

##### Notes

**Figure 1.**
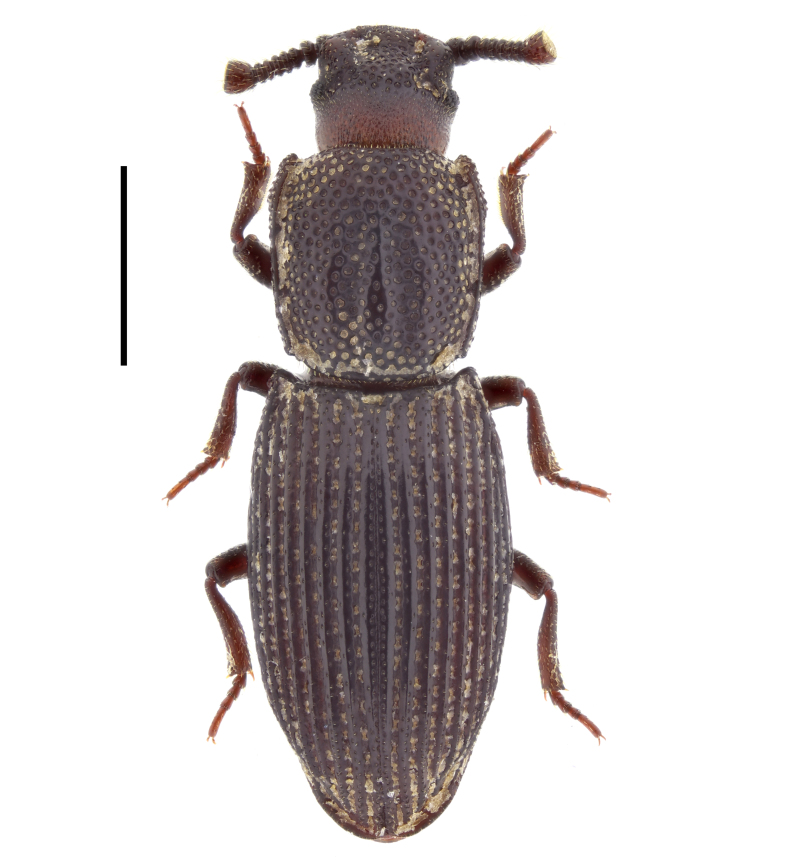
*Pycnomerus sulcicollis* (Germar, 1823) from Sarnena Gora Mountains, Bulgaria (female, BFUS-COL000738). Scale bar: 1 mm.

In Bulgaria, this species has been recorded from Emine Cape (Palm (1966): 13, as *Dechomus sulcicollis*), from the vicinity of Kamchia River mouth (Black Sea coast) (Dajoz (1971): 100, as *Dechomus sulcicollis*) and from Belasitsa Mountains (Guéorguiev (2011): 13). In the present study, we report this species from Strandzha, Belasitsa and Sarnena Gora Mountains (Fig. 1).

#### Pycnomerus terebrans

(G.-A. Olivier, 1790)

895F1FB9-76FF-5A90-B412-12149267F196

##### Materials

- **Type status:** Other material. **Occurrence:** catalogNumber: BFUS-COL000755; recordedBy: Denis Gradinarov; individualCount: 1; sex: male; occurrenceID: 783D4245-1CD0-5CF9-8FA4-298F4A1E46C9; **Location:** country: Bulgaria; stateProvince: Sofia City; municipality: Stolichna; locality: Sofia City, Borisova Gradina Park, near Park Hotel "Moskva"; **Identification:** identifiedBy: Denis Gradinarov; dateIdentified: 2021; **Event:** samplingProtocol: under pine bark; verbatimEventDate: 27.iv.1999; habitat: mixed forest
- **Type status:** Other material. **Occurrence:** catalogNumber: BFUS-COL000756; recordedBy: Denis Gradinarov; individualCount: 1; sex: female; occurrenceID: E54AFD85-732A-5B2C-A1E5-D579B6E3F701; **Location:** country: Bulgaria; stateProvince: Sofia City; municipality: Stolichna; locality: Sofia City, Borisova Gradina Park, near Park Hotel "Moskva"; **Identification:** identifiedBy: Denis Gradinarov; dateIdentified: 2021; **Event:** samplingProtocol: under pine bark; verbatimEventDate: 27.iv.1999; habitat: mixed forest
- **Type status:** Other material. **Occurrence:** catalogNumber: BFUS-COL000757; recordedBy: Denis Gradinarov; individualCount: 1; sex: specimen; occurrenceID: D64500A4-B5CD-5E67-84EE-B784766094B5; **Location:** country: Bulgaria; stateProvince: Sofia City; municipality: Stolichna; locality: Sofia City, Borisova Gradina Park, near Park Hotel "Moskva"; **Identification:** identifiedBy: Denis Gradinarov; dateIdentified: 2021; **Event:** samplingProtocol: under pine bark; verbatimEventDate: 27.iv.1999; habitat: mixed forest
- **Type status:** Other material. **Occurrence:** catalogNumber: BFUS-COL000758; recordedBy: Denis Gradinarov; individualCount: 1; sex: specimen; occurrenceID: 874C599B-B9C3-5BF9-9A0E-FC32B8648C60; **Location:** country: Bulgaria; stateProvince: Sofia City; municipality: Stolichna; locality: Sofia City, Borisova Gradina Park, near Park Hotel "Moskva"; **Identification:** identifiedBy: Denis Gradinarov; dateIdentified: 2021; **Event:** samplingProtocol: under pine bark; verbatimEventDate: 27.iv.1999; habitat: mixed forest
- **Type status:** Other material. **Occurrence:** catalogNumber: BFUS-COL000759; recordedBy: Denis Gradinarov; individualCount: 1; sex: specimen; occurrenceID: BEAA2B87-1F2A-5413-92C6-61FE0059EFC3; **Location:** country: Bulgaria; stateProvince: Sofia City; municipality: Stolichna; locality: Sofia City, Borisova Gradina Park, near Park Hotel "Moskva"; **Identification:** identifiedBy: Denis Gradinarov; dateIdentified: 2021; **Event:** samplingProtocol: under pine bark; verbatimEventDate: 27.iv.1999; habitat: mixed forest
- **Type status:** Other material. **Occurrence:** catalogNumber: BFUS-COL000760; recordedBy: Denis Gradinarov; individualCount: 1; sex: specimen; occurrenceID: E3C3E059-F271-5EDE-B73F-7C92E05B610D; **Location:** country: Bulgaria; stateProvince: Sofia City; municipality: Stolichna; locality: Sofia City, Borisova Gradina Park, near Park Hotel "Moskva"; **Identification:** identifiedBy: Denis Gradinarov; dateIdentified: 2021; **Event:** samplingProtocol: under pine bark; verbatimEventDate: 27.iv.1999; habitat: mixed forest
- **Type status:** Other material. **Occurrence:** catalogNumber: BFUS-COL000761; recordedBy: Denis Gradinarov; individualCount: 1; sex: male; occurrenceID: 676A9DB6-7022-59DB-A8B9-B9B7B39F5CB9; **Location:** country: Bulgaria; stateProvince: Sofia City; municipality: Stolichna; locality: Sofia City, Borisova Gradina Park; verbatimElevation: 600 m; decimalLatitude: 42.678083; decimalLongitude: 23.340583; geodeticDatum: WGS84; **Identification:** identifiedBy: Denis Gradinarov; dateIdentified: 2022; **Event:** samplingProtocol: under pine bark; verbatimEventDate: 15.vi.2021; habitat: mixed forest
- **Type status:** Other material. **Occurrence:** catalogNumber: BFUS-COL000762; recordedBy: Denis Gradinarov; individualCount: 1; sex: specimen; occurrenceID: CC508DFB-F1CB-5882-84B3-A46DDE5C78F6; **Location:** country: Bulgaria; stateProvince: Sofia City; municipality: Stolichna; locality: Sofia City, Borisova Gradina Park; verbatimElevation: 600 m; decimalLatitude: 42.678083; decimalLongitude: 23.340583; geodeticDatum: WGS84; **Identification:** identifiedBy: Denis Gradinarov; dateIdentified: 2022; **Event:** samplingProtocol: under pine bark; verbatimEventDate: 15.vi.2021; habitat: mixed forest
- **Type status:** Other material. **Occurrence:** catalogNumber: BFUS-COL000763; recordedBy: Denis Gradinarov; individualCount: 1; sex: specimen; occurrenceID: B0E165A7-5705-54E1-826F-B8D9BACC4995; **Location:** country: Bulgaria; stateProvince: Sofia City; municipality: Stolichna; locality: Sofia City, Borisova Gradina Park; verbatimElevation: 602 m; decimalLatitude: 42.678183; decimalLongitude: 23.340083; geodeticDatum: WGS84; **Identification:** identifiedBy: Denis Gradinarov; dateIdentified: 2026; **Event:** samplingProtocol: under bark of *Pinus sylvestris*; verbatimEventDate: 21.iii.2023; habitat: mixed forest
- **Type status:** Other material. **Occurrence:** catalogNumber: BFUS-COL000764; recordedBy: Denis Gradinarov; individualCount: 1; sex: specimen; occurrenceID: 0EF78271-3F68-5E87-B587-C0790132516A; **Location:** country: Bulgaria; stateProvince: Sofia City; municipality: Stolichna; locality: Sofia City, Borisova Gradina Park; verbatimElevation: 608 m; decimalLatitude: 42.678200; decimalLongitude: 23.340550; geodeticDatum: WGS84; **Identification:** identifiedBy: Denis Gradinarov; dateIdentified: 2026; **Event:** samplingProtocol: under bark of *Pinus sylvestris*; verbatimEventDate: 21.iii.2023; habitat: mixed forest
- **Type status:** Other material. **Occurrence:** catalogNumber: BFUS-COL000765; recordedBy: Denis Gradinarov; individualCount: 1; sex: male; occurrenceID: 5F0C1C1F-E106-583C-A082-B7BE53B9E648; **Location:** country: Bulgaria; stateProvince: Sofia City; municipality: Stolichna; locality: Sofia City, Borisova Gradina Park; verbatimElevation: 602 m; decimalLatitude: 42.678183; decimalLongitude: 23.340083; geodeticDatum: WGS84; **Identification:** identifiedBy: Denis Gradinarov; dateIdentified: 2026; **Event:** samplingProtocol: under bark of *Pinus nigra*; verbatimEventDate: 24.i.2025; habitat: mixed forest
- **Type status:** Other material. **Occurrence:** catalogNumber: BFUS-COL000766; recordedBy: Denis Gradinarov & Ognyan Sivilov; individualCount: 1; sex: male; occurrenceID: AD463E17-B1CD-5F87-8625-B5C70519F277; **Location:** country: Bulgaria; stateProvince: Sofia City; municipality: Stolichna; locality: Sofia City, Borisova Gradina Park; verbatimElevation: 602 m; decimalLatitude: 42.678183; decimalLongitude: 23.340083; geodeticDatum: WGS84; **Identification:** identifiedBy: Denis Gradinarov; dateIdentified: 2026; **Event:** samplingProtocol: under bark of *Pinus nigra*; verbatimEventDate: 28.i.2025; habitat: mixed forest
- **Type status:** Other material. **Occurrence:** catalogNumber: BFUS-COL000767; recordedBy: Denis Gradinarov & Ognyan Sivilov; individualCount: 1; sex: male; occurrenceID: A02C5659-1A13-5C9F-AA83-83598CC2C240; **Location:** country: Bulgaria; stateProvince: Sofia City; municipality: Stolichna; locality: Sofia City, Borisova Gradina Park; verbatimElevation: 602 m; decimalLatitude: 42.678183; decimalLongitude: 23.340083; geodeticDatum: WGS84; **Identification:** identifiedBy: Denis Gradinarov; dateIdentified: 2026; **Event:** samplingProtocol: under bark of *Pinus nigra*; verbatimEventDate: 28.i.2025; habitat: mixed forest
- **Type status:** Other material. **Occurrence:** catalogNumber: BFUS-COL000768; recordedBy: Denis Gradinarov; individualCount: 1; sex: male; occurrenceID: D8951E33-77E4-5BB8-BAE4-734342340FF6; **Location:** country: Bulgaria; stateProvince: Sofia City; municipality: Stolichna; locality: Sofia City, Borisova Gradina Park; verbatimElevation: 596 m; decimalLatitude: 42.680100; decimalLongitude: 23.339233; geodeticDatum: WGS84; **Identification:** identifiedBy: Denis Gradinarov; dateIdentified: 2026; **Event:** samplingProtocol: under bark of *Pinus sylvestris*; verbatimEventDate: 22.v.2025; habitat: mixed forest
- **Type status:** Other material. **Occurrence:** catalogNumber: BFUS-COL000769; recordedBy: Denis Gradinarov; individualCount: 1; sex: male; occurrenceID: 330AAF11-79A1-53B2-AFBA-6F009058EE4D; **Location:** country: Bulgaria; stateProvince: Sofia City; municipality: Stolichna; locality: Sofia City, Borisova Gradina Park; verbatimElevation: 596 m; decimalLatitude: 42.680100; decimalLongitude: 23.339233; geodeticDatum: WGS84; **Identification:** identifiedBy: Denis Gradinarov; dateIdentified: 2026; **Event:** samplingProtocol: under bark of *Pinus sylvestris*; verbatimEventDate: 22.v.2025; habitat: mixed forest
- **Type status:** Other material. **Occurrence:** catalogNumber: BFUS-COL000770; recordedBy: Denis Gradinarov; individualCount: 1; sex: male; occurrenceID: 56324A8F-34C9-5780-A8CE-FA23FF45A9FD; **Location:** country: Bulgaria; stateProvince: Sofia City; municipality: Stolichna; locality: Sofia City, Borisova Gradina Park; verbatimElevation: 596 m; decimalLatitude: 42.680100; decimalLongitude: 23.339233; geodeticDatum: WGS84; **Identification:** identifiedBy: Denis Gradinarov; dateIdentified: 2026; **Event:** samplingProtocol: under bark of *Pinus sylvestris*; verbatimEventDate: 22.v.2025; habitat: mixed forest
- **Type status:** Other material. **Occurrence:** catalogNumber: BFUS-COL000771; recordedBy: Denis Gradinarov; individualCount: 1; sex: female; occurrenceID: 350E2E3F-14A0-5359-97DE-66B6BEB0C08F; **Location:** country: Bulgaria; stateProvince: Sofia City; municipality: Stolichna; locality: Sofia City, Borisova Gradina Park; verbatimElevation: 596 m; decimalLatitude: 42.680100; decimalLongitude: 23.339233; geodeticDatum: WGS84; **Identification:** identifiedBy: Denis Gradinarov; dateIdentified: 2026; **Event:** samplingProtocol: under bark of *Pinus sylvestris*; verbatimEventDate: 22.v.2025; habitat: mixed forest
- **Type status:** Other material. **Occurrence:** catalogNumber: BFUS-COL000772; recordedBy: Denis Gradinarov; individualCount: 1; sex: female; occurrenceID: 6BA91968-3A03-562E-99F3-8B772516E0E8; **Location:** country: Bulgaria; stateProvince: Sofia City; municipality: Stolichna; locality: Sofia City, Borisova Gradina Park; verbatimElevation: 596 m; decimalLatitude: 42.680100; decimalLongitude: 23.339233; geodeticDatum: WGS84; **Identification:** identifiedBy: Denis Gradinarov; dateIdentified: 2026; **Event:** samplingProtocol: under bark of *Pinus sylvestris*; verbatimEventDate: 22.v.2025; habitat: mixed forest
- **Type status:** Other material. **Occurrence:** catalogNumber: BFUS-COL000773; recordedBy: Denis Gradinarov; individualCount: 1; sex: female; occurrenceID: C2639A85-448C-5C72-B8BA-78460DFE02E8; **Location:** country: Bulgaria; stateProvince: Sofia City; municipality: Stolichna; locality: Sofia City, Borisova Gradina Park; verbatimElevation: 596 m; decimalLatitude: 42.680100; decimalLongitude: 23.339233; geodeticDatum: WGS84; **Identification:** identifiedBy: Denis Gradinarov; dateIdentified: 2026; **Event:** samplingProtocol: under bark of *Pinus sylvestris*; verbatimEventDate: 22.v.2025; habitat: mixed forest
- **Type status:** Other material. **Occurrence:** catalogNumber: BFUS-COL000774; recordedBy: Denis Gradinarov; individualCount: 1; sex: female; occurrenceID: 00BFD72E-B95A-5394-AC66-EC5631523279; **Location:** country: Bulgaria; stateProvince: Sofia City; municipality: Stolichna; locality: Sofia City, Borisova Gradina Park; verbatimElevation: 596 m; decimalLatitude: 42.680100; decimalLongitude: 23.339233; geodeticDatum: WGS84; **Identification:** identifiedBy: Denis Gradinarov; dateIdentified: 2026; **Event:** samplingProtocol: under bark of *Pinus sylvestris*; verbatimEventDate: 22.v.2025; habitat: mixed forest
- **Type status:** Other material. **Occurrence:** catalogNumber: BFUS-COL000775; recordedBy: Denis Gradinarov; individualCount: 1; sex: female; occurrenceID: 400B16B7-2469-52E4-B757-F6EAC87A31FD; **Location:** country: Bulgaria; stateProvince: Sofia City; municipality: Stolichna; locality: Sofia City, Borisova Gradina Park; verbatimElevation: 596 m; decimalLatitude: 42.680100; decimalLongitude: 23.339233; geodeticDatum: WGS84; **Identification:** identifiedBy: Denis Gradinarov; dateIdentified: 2026; **Event:** samplingProtocol: under bark of *Pinus sylvestris*; verbatimEventDate: 22.v.2025; habitat: mixed forest
- **Type status:** Other material. **Occurrence:** catalogNumber: BFUS-COL000776; recordedBy: Denis Gradinarov & Ognyan Sivilov; individualCount: 1; sex: male; occurrenceID: 61E99387-8D8E-53AB-A169-24FF19103CD6; **Location:** country: Bulgaria; stateProvince: Sofia City; municipality: Stolichna; locality: Sofia City, Borisova Gradina Park; verbatimElevation: 596 m; decimalLatitude: 42.680100; decimalLongitude: 23.339233; geodeticDatum: WGS84; **Identification:** identifiedBy: Denis Gradinarov; dateIdentified: 2026; **Event:** samplingProtocol: under bark of *Pinus sylvestris*; verbatimEventDate: 27.v.2025; habitat: mixed forest
- **Type status:** Other material. **Occurrence:** catalogNumber: BFUS-COL000777; recordedBy: Denis Gradinarov & Ognyan Sivilov; individualCount: 1; sex: female; occurrenceID: 4FDFC4E6-BA25-5BBC-87B7-612D908BAFCA; **Location:** country: Bulgaria; stateProvince: Sofia City; municipality: Stolichna; locality: Sofia City, Borisova Gradina Park; verbatimElevation: 596 m; decimalLatitude: 42.680100; decimalLongitude: 23.339233; geodeticDatum: WGS84; **Identification:** identifiedBy: Denis Gradinarov; dateIdentified: 2026; **Event:** samplingProtocol: under bark of *Pinus nigra*; verbatimEventDate: 27.v.2025; habitat: mixed forest
- **Type status:** Other material. **Occurrence:** catalogNumber: BFUS-COL000778; recordedBy: Denis Gradinarov & Ognyan Sivilov; individualCount: 1; sex: female; occurrenceID: FFB40030-4526-58A1-BB83-E2C1FE5D6A2C; **Location:** country: Bulgaria; stateProvince: Sofia City; municipality: Stolichna; locality: Sofia City, Borisova Gradina Park; verbatimElevation: 596 m; decimalLatitude: 42.680100; decimalLongitude: 23.339233; geodeticDatum: WGS84; **Identification:** identifiedBy: Denis Gradinarov; dateIdentified: 2026; **Event:** samplingProtocol: under bark of *Pinus nigra*; verbatimEventDate: 27.v.2025; habitat: mixed forest
- **Type status:** Other material. **Occurrence:** catalogNumber: BFUS-COL000779; recordedBy: Denis Gradinarov; individualCount: 1; sex: female; occurrenceID: 390A651D-D8DE-5C48-8F4C-73EC16EDC5B6; **Location:** country: Bulgaria; stateProvince: Varna; municipality: Varna; locality: NE of Varna, University Botanical Garden; verbatimElevation: 59 m; decimalLatitude: 43.236967; decimalLongitude: 28.003917; geodeticDatum: WGS84; **Identification:** identifiedBy: Denis Gradinarov; dateIdentified: 2026; **Event:** samplingProtocol: at light; verbatimEventDate: 11.vii.2024; habitat: riverine forest

##### Distribution

Azerbaijan, Austria, Bosnia and Herzegovina, Bulgaria, Croatia, Czechia, France, Germany, Greece, Hungary, Italy, Poland, Portugal, Slovakia, Slovenia, Russia (Central and South European Territory), Switzerland, Ukraine, Serbia and Montenegro, European and Asian Türkiye, Iran (Schuh 2020); Moldova (Bacal 2023, Bacal and Buşmachiu 2025); Belgium (Thomaes et al. 2025).

##### Notes

**Figure 2.**
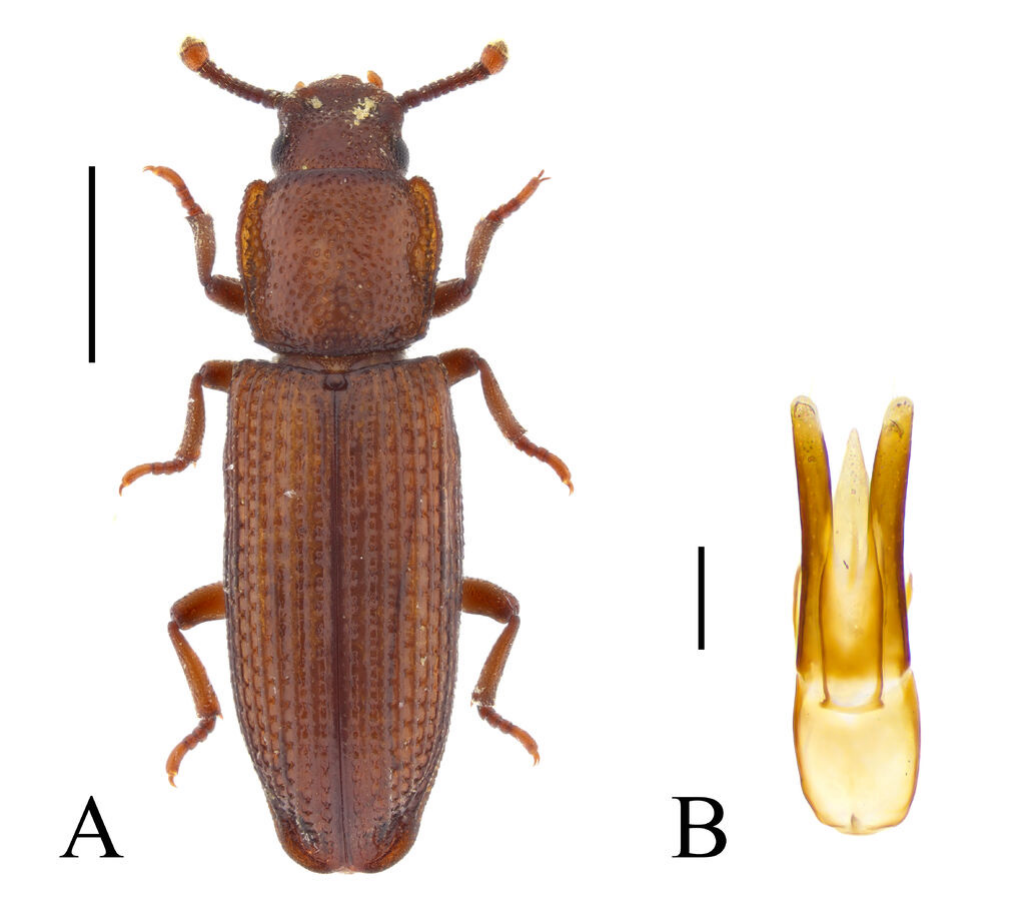
*Pycnomerus terebrans* (G.-A. Olivier, 1790) from Sofia City, Bulgaria: **A** male (BFUS-COL000768); **B** aedeagus of the same specimen. Scale bars: 1 mm (A); 0.1 mm (B).

In Bulgaria, this species has been recorded from Emine Cape (Palm (1966): 14), from the vicinity of Kamchia River mouth (Black Sea coast) (Dajoz (1971): 100) and from Belasitsa Mountains (Guéorguiev (2011): 13). Here, we provide data on the occurrence of *P. terebrans* in city parks of Sofia (Fig. 2) and Varna.

#### Colydiinae

Billberg, 1820

B51C1DC2-2D41-5627-B7CA-A0A874128F34

#### Colydiini

Billberg, 1820

1015BEDC-9188-5735-AE70-A8D843B5703C

#### Aulonium

Erichson, 1845

6EC561B7-3DB1-5851-B0CB-9C3D4EA6EDBA

#### Aulonium ruficorne

(A. G. Olivier, 1790)

26C84E24-70AE-5699-A0A9-012520478AD0

##### Materials

- **Type status:** Other material. **Occurrence:** catalogNumber: BFUS-COL000780; recordedBy: Denis Gradinarov & Yana Petrova; individualCount: 1; sex: male; occurrenceID: C9D0BD33-2645-5BDD-9409-DDD686290C16; **Location:** country: Bulgaria; stateProvince: Haskovo; municipality: Topolovgrad; locality: Sakar Mountains, SE of Ustrem Village; verbatimElevation: 215 m; decimalLatitude: 41.972583; decimalLongitude: 26.483117; geodeticDatum: WGS84; **Identification:** identifiedBy: Denis Gradinarov; dateIdentified: 2026; **Event:** samplingProtocol: under peeled pine bark; verbatimEventDate: 14.iv.2023; habitat: mixed forest

##### Distribution

Azerbaijan, Armenia, Bulgaria, Croatia, France, Germany, Greece, Hungary, Italy, North Macedonia, Romania, Spain, South European Territory of Russia, Ukraine, Serbia and Montenegro, European and Asian Türkiye, Iran, Israel, Lebanon, Turkmenistan, Algeria, Morocco, Tunisia (Schuh 2020); Portugal (Molina 2023).

##### Notes

**Figure 3.**
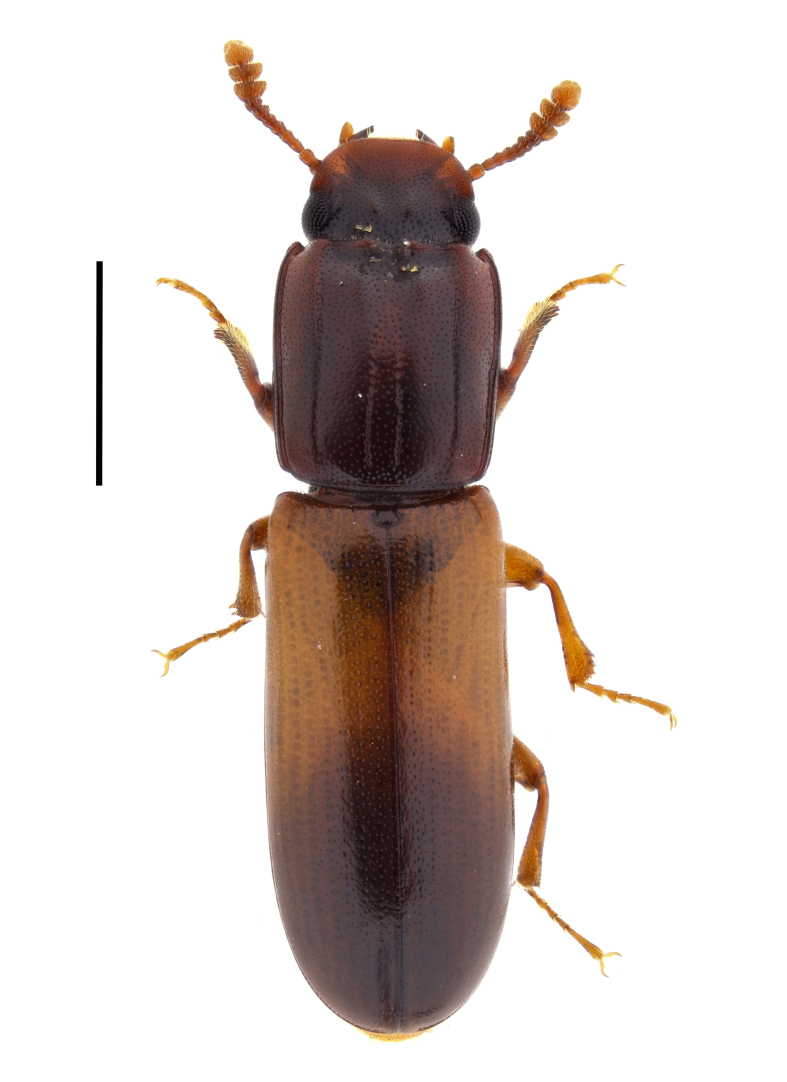
*Aulonium ruficorne* (G.-A. Olivier, 1790) from Sakar Mountains, Bulgaria (male, BFUS-COL000780). Scale bar: 1 mm.

This species has been previously reported for Bulgaria without exact locality (Ślipiński and Schuh 2008, Schuh 2020). In the present study, we report *A. ruficorne* from Sakar Mountains (Fig. 3) and, thus, confirm the presence of this species for the country.

#### Aulonium trisulcum

(Geoffroy, 1785)

2104AE3C-3BD8-5EF2-B632-82A7D4D6D81B

##### Materials

- **Type status:** Other material. **Occurrence:** catalogNumber: BFUS-COL000781; recordedBy: Ognyan Sivilov & Boyan Zlatkov; individualCount: 1; sex: male; occurrenceID: 975B1C35-87E5-5BA4-B1DD-8D96921A40E0; **Location:** country: Bulgaria; stateProvince: Blagoevgrad; municipality: Hadzhidimovo; locality: SE of Paril Village; verbatimElevation: 755 m; decimalLatitude: 41.432667; decimalLongitude: 23.700117; geodeticDatum: WGS84; **Identification:** identifiedBy: Denis Gradinarov; dateIdentified: 2026; **Event:** samplingProtocol: at light; verbatimEventDate: 17.vi.2013–18.vi.2013; habitat: riverine vegetation
- **Type status:** Other material. **Occurrence:** catalogNumber: BFUS-COL000782; recordedBy: Ognyan Sivilov & Boyan Zlatkov; individualCount: 1; sex: male; occurrenceID: 7E419537-05C7-59CD-85D5-9B450A3A812A; **Location:** country: Bulgaria; stateProvince: Blagoevgrad; municipality: Hadzhidimovo; locality: SE of Paril Village; verbatimElevation: 755 m; decimalLatitude: 41.432667; decimalLongitude: 23.700117; geodeticDatum: WGS84; **Identification:** identifiedBy: Denis Gradinarov; dateIdentified: 2026; **Event:** samplingProtocol: at light; verbatimEventDate: 17.vi.2013–18.vi.2013; habitat: riverine vegetation
- **Type status:** Other material. **Occurrence:** catalogNumber: BFUS-COL000783; recordedBy: Ognyan Sivilov & Boyan Zlatkov; individualCount: 1; sex: specimen; occurrenceID: AD72B4CD-E3DA-587A-8C4C-EA50D07CF643; **Location:** country: Bulgaria; stateProvince: Blagoevgrad; municipality: Hadzhidimovo; locality: SE of Paril Village; verbatimElevation: 755 m; decimalLatitude: 41.432667; decimalLongitude: 23.700117; geodeticDatum: WGS84; **Identification:** identifiedBy: Denis Gradinarov; dateIdentified: 2026; **Event:** samplingProtocol: at light; verbatimEventDate: 17.vi.2013–18.vi.2013; habitat: riverine vegetation
- **Type status:** Other material. **Occurrence:** catalogNumber: BFUS-COL000784; recordedBy: Ognyan Sivilov & Boyan Zlatkov; individualCount: 1; sex: female; occurrenceID: 8D88AE83-1C11-57EA-85BF-1C87D5F89EF9; **Location:** country: Bulgaria; stateProvince: Blagoevgrad; municipality: Petrich; locality: Sandanski-Petrich Valley, SE of Starchevo Village; verbatimElevation: 95 m; decimalLatitude: 41.46812; decimalLongitude: 23.26900; geodeticDatum: WGS84; **Identification:** identifiedBy: Denis Gradinarov; dateIdentified: 2026; **Event:** samplingProtocol: at light; verbatimEventDate: 20.vi.2013–21.vi.2013; habitat: riverine forest
- **Type status:** Other material. **Occurrence:** catalogNumber: BFUS-COL000785; recordedBy: Ognyan Sivilov & Boyan Zlatkov; individualCount: 1; sex: specimen; occurrenceID: 367EFFBA-6706-53ED-8B1A-F221FFCA4D6C; **Location:** country: Bulgaria; stateProvince: Varna; municipality: Dolni Chiflik; locality: Black Sea coast, E of Novo Oryahovo Village; verbatimElevation: 8 m; decimalLatitude: 42.99181; decimalLongitude: 27.89002; geodeticDatum: WGS84; **Identification:** identifiedBy: Denis Gradinarov; dateIdentified: 2026; **Event:** samplingProtocol: at light; verbatimEventDate: 03.vii.2013–04.vii.2013; habitat: sandy beach next to longos forest
- **Type status:** Other material. **Occurrence:** catalogNumber: BFUS-COL000786; recordedBy: Denis Gradinarov & Yana Petrova; individualCount: 1; sex: specimen; occurrenceID: C9B3867A-0CBE-50E8-9DB3-26BF4E5108FF; **Location:** country: Bulgaria; stateProvince: Pernik; municipality: Zemen; locality: Zemen Gorge, SW of Zemen; verbatimElevation: 593 m; decimalLatitude: 42.471056; decimalLongitude: 22.731361; geodeticDatum: WGS84; **Identification:** identifiedBy: Denis Gradinarov; dateIdentified: 2021; **Event:** samplingProtocol: at light; verbatimEventDate: 05.vii.2015
- **Type status:** Other material. **Occurrence:** catalogNumber: BFUS-COL000787; recordedBy: Ognyan Sivilov & Boyan Zlatkov; individualCount: 1; sex: female; occurrenceID: BB0E230C-901E-5B63-9F27-69E42075263A; **Location:** country: Bulgaria; stateProvince: Blagoevgrad; municipality: Sandanski; locality: Pirin Mountains, SW of Lilyanovo Village; verbatimElevation: 470 m; decimalLatitude: 41.612467; decimalLongitude: 23.311917; geodeticDatum: WGS84; **Identification:** identifiedBy: Denis Gradinarov; dateIdentified: 2026; **Event:** samplingProtocol: at light; verbatimEventDate: 27.vi.2019–28.vi.2019; habitat: deciduous grove
- **Type status:** Other material. **Occurrence:** catalogNumber: BFUS-COL000788; recordedBy: Ognyan Sivilov & Boyan Zlatkov; individualCount: 1; sex: female; occurrenceID: EDAF9236-2F23-5756-9128-6F641E61E4D7; **Location:** country: Bulgaria; stateProvince: Blagoevgrad; municipality: Sandanski; locality: Sandanski-Petrich Valley, E of Novo Hodzhovo Village; verbatimElevation: 124 m; decimalLatitude: 41.40721; decimalLongitude: 23.40666; geodeticDatum: WGS84; **Identification:** identifiedBy: Denis Gradinarov; dateIdentified: 2026; **Event:** samplingProtocol: at light; verbatimEventDate: 30.vi.2019–01.vii.2019; habitat: riverine forest
- **Type status:** Other material. **Occurrence:** catalogNumber: BFUS-COL000789; recordedBy: Ognyan Sivilov & Boyan Zlatkov; individualCount: 1; sex: specimen; occurrenceID: 1673B0BB-B411-5BAB-8F7C-481C177B256E; **Location:** country: Bulgaria; stateProvince: Blagoevgrad; municipality: Kresna; locality: Kresnenski Prolom Gorge, Sheitan Dere; verbatimElevation: 205 m; decimalLatitude: 41.760583; decimalLongitude: 23.154583; geodeticDatum: WGS84; **Identification:** identifiedBy: Denis Gradinarov; dateIdentified: 2026; **Event:** samplingProtocol: at light; verbatimEventDate: 01.vii.2019–02.vii.2019
- **Type status:** Other material. **Occurrence:** catalogNumber: BFUS-COL000790; recordedBy: Denis Gradinarov; individualCount: 1; sex: specimen; occurrenceID: FCE55DCA-2A4A-5ECB-B0D6-A01DA0371385; **Location:** country: Bulgaria; stateProvince: Haskovo; municipality: Topolovgrad; locality: Sakar Mountains, W of Ustrem Village; verbatimElevation: 101 m; decimalLatitude: 42.024167; decimalLongitude: 26.451550; geodeticDatum: WGS84; **Identification:** identifiedBy: Denis Gradinarov; dateIdentified: 2026; **Event:** samplingProtocol: at light; verbatimEventDate: 28.vi.2022; habitat: riverine vegetation
- **Type status:** Other material. **Occurrence:** catalogNumber: BFUS-COL000791; recordedBy: Denis Gradinarov; individualCount: 1; sex: female; occurrenceID: 5453AEF3-6D75-55B0-ABA0-59737A7ED0EB; **Location:** country: Bulgaria; stateProvince: Varna; municipality: Varna; locality: NE of Varna, University Botanical Garden; verbatimElevation: 47 m; decimalLatitude: 43.233750; decimalLongitude: 28.003617; geodeticDatum: WGS84; **Identification:** identifiedBy: Denis Gradinarov; dateIdentified: 2026; **Event:** samplingProtocol: at light; verbatimEventDate: 13.vii.2024; habitat: meadow

##### Distribution

Armenia, Austria, Belgium, Bosnia and Herzegovina, Czechia, France, Germany, Great Britain, Hungary, Italy, The Netherlands, North Macedonia, Norway, Poland, Russia (South, Central and North European Territory), Slovakia, Sweden, Switzerland, Ukraine, Iran (Schuh 2020); Spain, Portugal (Molina 2023); Luxembourg (Gerend 2023); Moldova (Bacal and Buşmachiu 2025); Bulgaria (present study).

##### Notes

**Figure 4.**
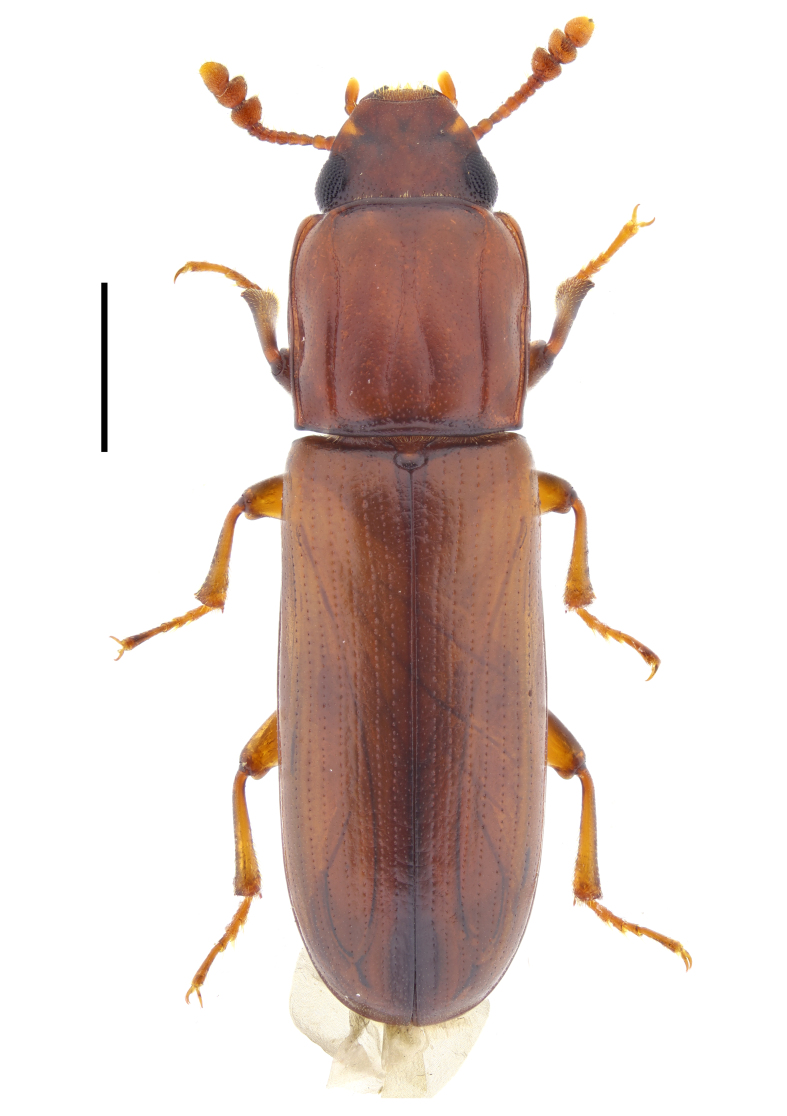
*Aulonium trisulcum* (Geoffroy, 1785) from Varna, Bulgaria (female, BFUS-COL000791). Scale bar: 1 mm.

This species has been previously reported for Bulgaria without exact locality by Anonymous (1907): 300, as *Aulonium sulcatum* [(G.-A. Olivier, 1790)]. The species is omitted for Bulgaria in both editions of the Catalogue of Palaearctic Coleoptera (Ślipiński and Schuh 2008; Schuh 2020). Here, we report *A. trisulcum* from several localities in south-western Bulgaria, from Sakar Mountains and from the Black Sea coast (Fig. 4) and, thus, confirm the presence of this species for the country. In our study, all the specimens were collected at light.

#### Colydium

Fabricius, 1792

8931CE57-0F9B-540C-B8D4-3B3A8C46F5F5

#### Colydium elongatum

(Fabricius, 1787)

90555794-142A-594B-8A3C-423559C821BE

##### Distribution

Azerbaijan, Albania, Armenia, Austria, Belgium, Bosnia and Herzegovina, Bulgaria, Belarus, Croatia, Czechia, Denmark, Finland, France, Great Britain, Germany, Greece, Hungary, Italy, Lithuania, North Macedonia, The Netherlands, Poland, Romania, Russia (European and Asian Territory), Slovakia, Slovenia, Spain, Sweden, Switzerland, Ukraine, Serbia and Montenegro, European and Asian Türkiye, Cyprus, Iran, Syria, Algeria, Morocco, Tunisia (Schuh 2020).

##### Notes

**Figure 5.**
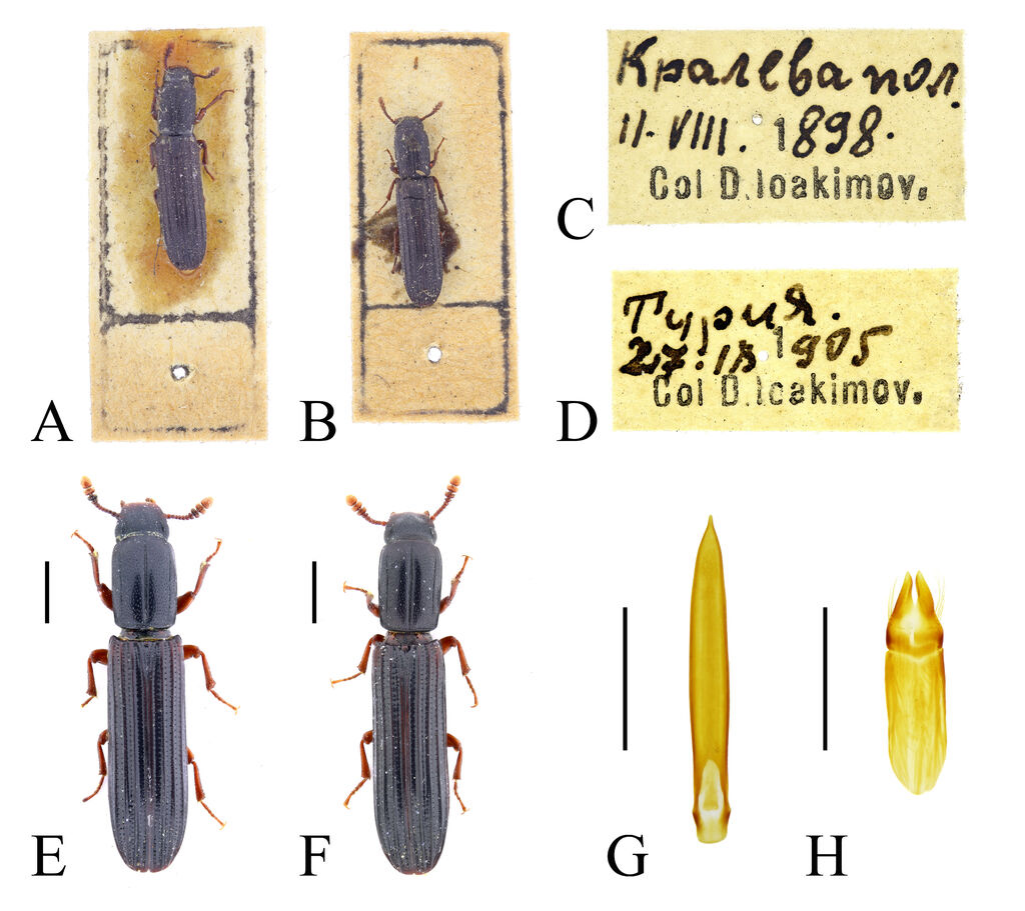
Specimens of *Colydium noblecourti* Parmain, Eckelt & Schuh, 2024 preserved in the historical collection of Dimitar Joakimov, NMNHS. **A** male from Kraleva Polyana locality (specimen 1); **B** female from Turiya locality (specimen 2); **C** original label (specimen 1); **D** original label (specimen 2); **E** specimen 1 (re-mounted); **F** specimen 2 (re-mounted); **G** median lobe (specimen 1); **H** tegmen (specimen 1). Scale bars: 1 mm (E, F); 0.5 mm (G, H).

In Bulgaria, *C. elongatum* has been previously recorded from Sarnena Sredna Gora Mountains (Joakimov (1904): 16), from the vicinity of Ropotamo River mouth (Black Sea coast) (Dajoz (1971): 100), from Kresnenski Prolom Gorge, Maslen Nos Cape, Strandzha Mountains (Węgrzynowicz 1999: 287) and from north-eastern Bulgaria (without exact localities) (Parmain et al. (2024): 300 (map)). *Colydium elongatum* is similar in habitus to recently described *C. noblecourti* Parmain, Eckelt & Schuh, 2024 and some of the earlier records may, in fact, refer to the second species. Both specimens found in the historical collection of Dimitar Joakimov (NMNHS) and previously identified as *C. elongatum*, after revision of the identification turned out to be *C. noblecourti* (Fig. 5).

#### Colydium filiforme

Fabricius, 1792

C2F4817F-8279-5B4B-A058-6A5997DE3A67

##### Distribution

Azerbaijan, Austria, Belgium, Bosnia and Herzegovina, Bulgaria, Belarus, Croatia, Czechia, France, Germany, Georgia, Greece, Hungary, Italy, Latvia, Liechtenstein, Norway, Poland, Russia (South European and Asian Territory), Slovakia, Spain, Sweden, Switzerland, Ukraine, Iran (Schuh 2020); Central European Territory of Russia (Egorov et al. 2020); Asian Türkiye (Harman and Avcı 2023); Serbia (Marczak and Pepłowska-Marczak 2025).

##### Notes

In Bulgaria, this species has been recorded from Slanchev Bryag seaside resort (Palm 1966: 22) and Belasitsa Mountains (Guéorguiev 2011: 13).

#### Colydium noblecourti

Parmain, Eckelt & Schuh, 2024

E0587C6F-B297-5E55-8122-EE366863DA37

##### Materials

- **Type status:** Other material. **Occurrence:** recordedBy: Dimitar Joakimov; individualCount: 1; sex: male; previousIdentifications: *Colydium elongatum* F.; occurrenceID: EF75C028-3DF3-551F-A91C-02CF522A2F07; **Location:** country: Bulgaria; locality: Kraleva Pol[yana]; verbatimLocality: Kraleva pol. [in Bulgarien]; **Identification:** identifiedBy: Denis Gradinarov; dateIdentified: 2026; **Event:** verbatimEventDate: II–VIII.1898
- **Type status:** Other material. **Occurrence:** recordedBy: Dimitar Joakimov; individualCount: 1; sex: female; previousIdentifications: *Colydium elongatum* F.; occurrenceID: 7489FC00-B145-51F7-B7FE-D61CA16C1592; **Location:** country: Bulgaria; stateProvince: Stara Zagora; municipality: Pavel Banya; locality: [Sarnena Gora Mountains], Turiya Village; verbatimLocality: Turiya. [in Bulgarien]; **Identification:** identifiedBy: Denis Gradinarov; dateIdentified: 2026; **Event:** verbatimEventDate: 27.IX.1905
- **Type status:** Other material. **Occurrence:** catalogNumber: BFUS-COL000792; recordedBy: Ognyan Sivilov; individualCount: 1; sex: male; occurrenceID: 24DAE36B-7B31-5552-875A-9FE2BBD573AE; **Location:** country: Bulgaria; stateProvince: Sliven; municipality: Tvarditsa; locality: Central Stara Planina Mountains, near Bukovets Hut; verbatimElevation: 1052 m; decimalLatitude: 42.789225; decimalLongitude: 25.890651; geodeticDatum: WGS84; **Identification:** identifiedBy: Denis Gradinarov; dateIdentified: 2026; **Event:** samplingProtocol: at night on tree trunks; verbatimEventDate: 27.viii.2009; habitat: beech forest

##### Distribution

Austria, Andorra, Bosnia, Croatia, Czechia, France, Germany, Iran, Italy, Slovakia, Slovenia, Spain, Asian part of Türkiye (Parmain et al. 2024); Serbia (Marczak and Pepłowska-Marczak 2025); Russia (Kaliningrad Oblast) (Alekseev 2025); Belgium (Thomaes et al. 2025); Bulgaria (present study).

##### Notes

**Figure 6.**
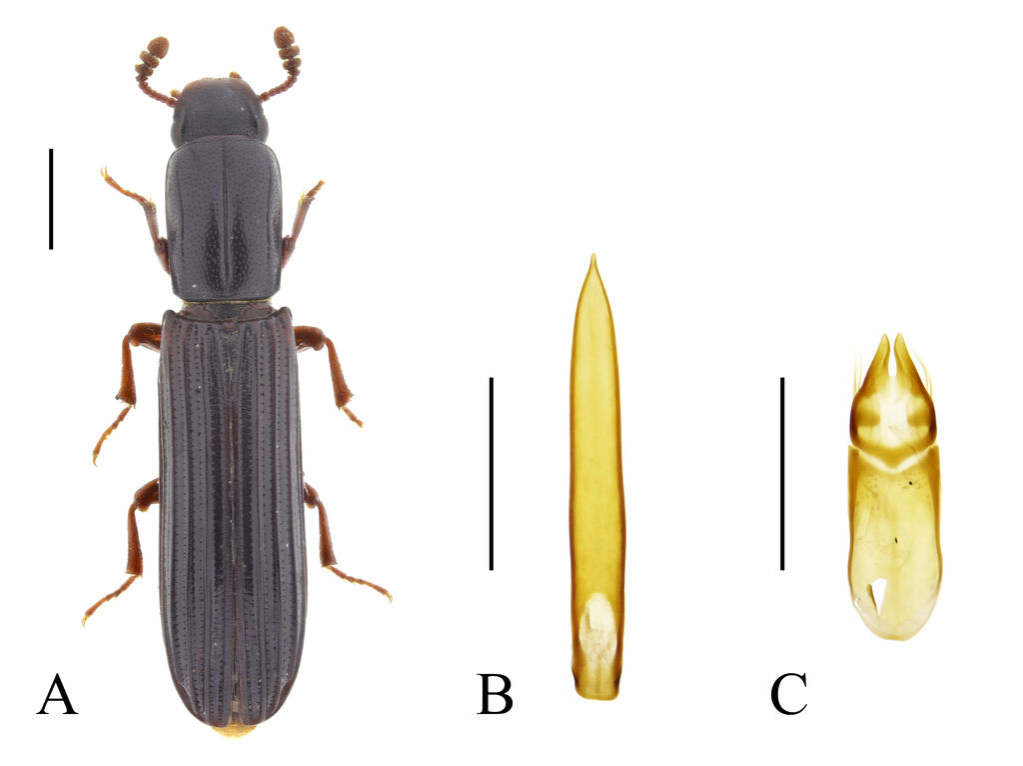
*Colydium noblecourti* Parmain, Eckelt & Schuh, 2024 from Central Stara Planina Mountains, Bulgaria: **A** male (BFUS-COL000792); **B** median lobe of the same specimen; **C** tegmen of the same specimen. Scale bars: 1 mm (A); 0.5 mm (B, C).

In the present study, we report *C. noblecourti* from "Kraleva polyana" (probably, Kraleva livada locality near Kameno Pole Village, in north-eastern Bulgaria), from Sarnena Gora Mountains and from Central Stara Planina Mountains (Fig. 5; Fig. 6). New records for Bulgaria.

#### Orthocerini

Blanchard, 1845 (1820)

BDE5B3F5-03EF-5C77-9FC5-8B78D45B33FB

#### Orthocerus

Latreille, 1796

3CA33456-CDEC-5F28-A86D-D62FC2C79ECD

#### Orthocerus clavicornis

(Linnaeus, 1758)

3B898565-9E05-591D-9910-751E6E8CD9F2

##### Materials

- **Type status:** Other material. **Occurrence:** catalogNumber: BFUS-COL000793; recordedBy: Nikolay Simov; individualCount: 1; sex: female; occurrenceID: 7C004743-F2BF-527E-884D-51D5374A1D08; **Location:** country: Bulgaria; stateProvince: Blagoevgrad; municipality: Sandanski; locality: Alibotush Mt [= Slavyanka Mts], Souhoto Ezero, near Gotsev Vrah Peak; verbatimElevation: 2065 m; **Identification:** identifiedBy: Denis Gradinarov; dateIdentified: 2020; **Event:** samplingProtocol: pitfall traps; verbatimEventDate: 5.vii.2006–10.viii.2006

##### Distribution

Austria, Belgium, Bosnia and Herzegovina, Belarus, Croatia, Czechia, Denmark, Finland, France, Germany, Great Britain, Hungary, Latvia, Liechtenstein, Luxembourg, The Netherlands, Norway, Italy, Ireland, Poland, Romania, Russia (Central European Territory, East Siberia, Far East), Slovakia, Spain, Sweden, Switzerland, Ukraine, Serbia and Montenegro, “Caucasus”, Asian Türkiye, Mongolia (Schuh 2020); Iran (Otero and Ghahari 2020); Belgium (Thomaes et al. 2025); Bulgaria (Angelov 1968a; present study).

##### Notes

**Figure 7.**
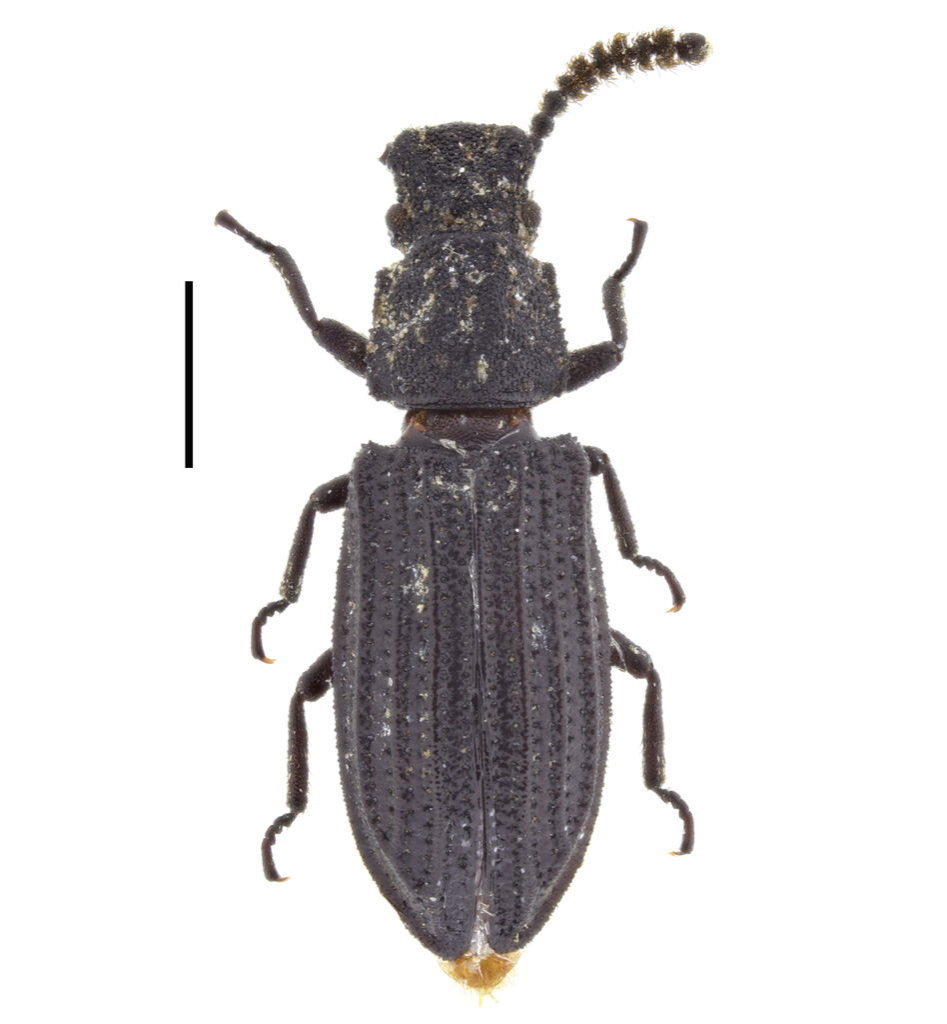
*Orthocerus clavicornis* (Linnaeus, 1758) from Slavyanka Mountains, Bulgaria (female, BFUS-COL000793). Scale bar: 1 mm.

In Bulgaria, this species has been reported from several localities in Western Rhodopes Mountains and from Izdremets Peak in Stara Planina Mountains (Angelov 1968a: 99). The species is omitted for Bulgaria in both editions of the Catalogue of Palaearctic Coleoptera (Ślipiński and Schuh 2008; Schuh 2020). Here, we report *O. clavicornis* from Slavyanka Mountains (Fig. 7) and confirm the presence of this species in Bulgaria.

#### Rhopalocerini

Reitter, 1911

146D9104-2675-5AB0-A441-678AADC18C4B

#### Rhopalocerus

W. Redtenbacher, 1842

D535C9C3-1190-5943-8EB9-9E5DC790F83A

#### Rhopalocerus rondanii

(A. Villa & J. B. Villa, 1833)

CD36217A-D407-5711-B12C-3FCF9A56C8DB

##### Distribution

Austria, Bulgaria, Croatia, Czechia, Germany, Greece, Hungary, Italy, Portugal, Romania, Slovakia, Ukraine, Serbia and Montenegro (Schuh 2020); Iran (Farashiani et al. 2022); Moldova (Bacal 2023, Bacal and Buşmachiu 2025).

##### Notes

The species has been recorded for Bulgaria without exact locality (Ślipiński and Burakowski 1988: 92, Ślipiński and Schuh 2008: 80, Schuh 2020: 69).

#### Synchitini

Erichson, 1845

8032484C-375A-5EB6-A488-CFCDA7615EB6

#### Bitoma

Herbst, 1793

FD3349BB-0323-5374-9644-A293C3A1B38B

#### Bitoma crenata

(Fabricius, 1775)

FF838F6E-5C9C-5E70-AC10-0869E52BC764

##### Materials

- **Type status:** Other material. **Occurrence:** catalogNumber: BFUS-COL000794; recordedBy: Stoyan Dimitrov; individualCount: 1; sex: specimen; occurrenceID: 3E0F1F2C-CB4E-5F64-9339-76B85D8607C3; **Location:** country: Bulgaria; stateProvince: Pernik; municipality: Zemen; locality: [Zemen Gorge], near Zemen; verbatimLocality: near Zemen; **Identification:** identifiedBy: Denis Gradinarov; dateIdentified: 2026; **Event:** samplingProtocol: under bark; verbatimEventDate: 15.iv.2000
- **Type status:** Other material. **Occurrence:** catalogNumber: BFUS-COL000795; recordedBy: Stoyan Dimitrov; individualCount: 1; sex: specimen; occurrenceID: EC7A4D03-68AE-5048-9A6B-B28BF2116FD5; **Location:** country: Bulgaria; stateProvince: Pernik; municipality: Zemen; locality: [Zemen Gorge], near Zemen; verbatimLocality: near Zemen; **Identification:** identifiedBy: Denis Gradinarov; dateIdentified: 2026; **Event:** samplingProtocol: under bark; verbatimEventDate: 15.iv.2000
- **Type status:** Other material. **Occurrence:** catalogNumber: BFUS-COL000796; recordedBy: Yana Petrova, Denis Gradinarov & Evgeni Chehlarov; individualCount: 1; sex: specimen; occurrenceID: 4090C09F-759D-5027-AFB2-936681F90E25; **Location:** country: Bulgaria; stateProvince: Kyustendil; municipality: Kyustendil; locality: Osogovska Planina Mountains, near Trite buki Hut; verbatimElevation: 1550 m; **Identification:** identifiedBy: Denis Gradinarov; dateIdentified: 2020; **Event:** verbatimEventDate: 22.viii.2003; habitat: beech forest
- **Type status:** Other material. **Occurrence:** catalogNumber: BFUS-COL000797; recordedBy: Yana Petrova, Denis Gradinarov & Evgeni Chehlarov; individualCount: 1; sex: specimen; occurrenceID: AA3C1630-A03B-5931-8E41-629B3704E499; **Location:** country: Bulgaria; stateProvince: Kyustendil; municipality: Kyustendil; locality: Osogovska Planina Mountains, near Trite buki Hut; verbatimElevation: 1550 m; **Identification:** identifiedBy: Denis Gradinarov; dateIdentified: 2020; **Event:** verbatimEventDate: 22.viii.2003; habitat: beech forest
- **Type status:** Other material. **Occurrence:** catalogNumber: BFUS-COL000798; recordedBy: Yana Petrova, Denis Gradinarov & Evgeni Chehlarov; individualCount: 1; sex: specimen; occurrenceID: F7EBD4BB-DDBA-57A1-B296-2CEDFE16E36F; **Location:** country: Bulgaria; stateProvince: Kyustendil; municipality: Kyustendil; locality: Osogovska Planina Mountains, near Trite buki Hut; verbatimElevation: 1550 m; **Identification:** identifiedBy: Denis Gradinarov; dateIdentified: 2020; **Event:** verbatimEventDate: 22.viii.2003; habitat: beech forest
- **Type status:** Other material. **Occurrence:** catalogNumber: BFUS-COL000799; recordedBy: Yana Petrova, Denis Gradinarov & Evgeni Chehlarov; individualCount: 1; sex: specimen; occurrenceID: 57087641-23D9-56DC-8413-166042393377; **Location:** country: Bulgaria; stateProvince: Kyustendil; municipality: Kyustendil; locality: Osogovska Planina Mountains, near Trite buki Hut; verbatimElevation: 1550 m; **Identification:** identifiedBy: Denis Gradinarov; dateIdentified: 2020; **Event:** verbatimEventDate: 22.viii.2003; habitat: beech forest
- **Type status:** Other material. **Occurrence:** catalogNumber: BFUS-COL000800; recordedBy: Ognyan Sivilov; individualCount: 1; sex: specimen; occurrenceID: D186FD7D-D947-591F-B139-A3CCEAD45B2F; **Location:** country: Bulgaria; stateProvince: Blagoevgrad; municipality: Satovcha; locality: Western Rhodopes Mountains, E of Dolen Village; verbatimElevation: 887 m; decimalLatitude: 41.622292; decimalLongitude: 23.947836; geodeticDatum: WGS84; **Identification:** identifiedBy: Denis Gradinarov; dateIdentified: 2026; **Event:** samplingProtocol: in oak stump; verbatimEventDate: 18.x.2011; habitat: mixed forest
- **Type status:** Other material. **Occurrence:** catalogNumber: BFUS-COL000801; recordedBy: Ognyan Sivilov; individualCount: 1; sex: specimen; occurrenceID: 99EB2DB0-4701-5DE0-8405-70EB47DBC7E0; **Location:** country: Bulgaria; stateProvince: Blagoevgrad; municipality: Satovcha; locality: Western Rhodopes Mountains, E of Dolen Village; verbatimElevation: 887 m; decimalLatitude: 41.622292; decimalLongitude: 23.947836; geodeticDatum: WGS84; **Identification:** identifiedBy: Denis Gradinarov; dateIdentified: 2026; **Event:** samplingProtocol: in oak stump; verbatimEventDate: 18.x.2011; habitat: mixed forest
- **Type status:** Other material. **Occurrence:** catalogNumber: BFUS-COL000802; recordedBy: Ognyan Sivilov; individualCount: 1; sex: specimen; occurrenceID: 96C2D2ED-A7FF-5AC2-B6CD-87B522D9EC87; **Location:** country: Bulgaria; stateProvince: Blagoevgrad; municipality: Satovcha; locality: Western Rhodopes Mountains, E of Dolen Village; verbatimElevation: 887 m; decimalLatitude: 41.622292; decimalLongitude: 23.947836; geodeticDatum: WGS84; **Identification:** identifiedBy: Denis Gradinarov; dateIdentified: 2026; **Event:** samplingProtocol: in oak stump; verbatimEventDate: 18.x.2011; habitat: mixed forest
- **Type status:** Other material. **Occurrence:** catalogNumber: BFUS-COL000803; recordedBy: Denis Gradinarov; individualCount: 1; sex: male; occurrenceID: DA421E1B-9BAA-5864-B4EE-4D531EC05D1A; **Location:** country: Bulgaria; stateProvince: Pernik; municipality: Zemen; locality: Zemen Valley; verbatimElevation: 587 m; decimalLatitude: 42.472250; decimalLongitude: 22.737667; geodeticDatum: WGS84; **Identification:** identifiedBy: Denis Gradinarov; dateIdentified: 2026; **Event:** samplingProtocol: under bark of *Populus nigra*; verbatimEventDate: 03.xi.2012; habitat: riverine forest
- **Type status:** Other material. **Occurrence:** catalogNumber: BFUS-COL000804; recordedBy: Denis Gradinarov; individualCount: 1; sex: male; occurrenceID: 631DE704-E427-5456-9EC7-4ADC9BC21E37; **Location:** country: Bulgaria; stateProvince: Pernik; municipality: Zemen; locality: Zemen Valley; verbatimElevation: 587 m; decimalLatitude: 42.472250; decimalLongitude: 22.737667; geodeticDatum: WGS84; **Identification:** identifiedBy: Denis Gradinarov; dateIdentified: 2026; **Event:** samplingProtocol: under bark of *Populus nigra*; verbatimEventDate: 03.xi.2012; habitat: riverine forest
- **Type status:** Other material. **Occurrence:** catalogNumber: BFUS-COL000805; recordedBy: Denis Gradinarov; individualCount: 1; sex: female; occurrenceID: 8ABE7269-B9B8-5F27-BDED-E4F6CE63186E; **Location:** country: Bulgaria; stateProvince: Pernik; municipality: Zemen; locality: Zemen Valley; verbatimElevation: 587 m; decimalLatitude: 42.472250; decimalLongitude: 22.737667; geodeticDatum: WGS84; **Identification:** identifiedBy: Denis Gradinarov; dateIdentified: 2026; **Event:** samplingProtocol: under bark of *Populus nigra*; verbatimEventDate: 03.xi.2012; habitat: riverine forest
- **Type status:** Other material. **Occurrence:** catalogNumber: BFUS-COL000806; recordedBy: Denis Gradinarov; individualCount: 1; sex: female; occurrenceID: BFC69074-0F18-5E97-B0A3-9C6EC9CC3D43; **Location:** country: Bulgaria; stateProvince: Pernik; municipality: Zemen; locality: Zemen Valley; verbatimElevation: 587 m; decimalLatitude: 42.472250; decimalLongitude: 22.737667; geodeticDatum: WGS84; **Identification:** identifiedBy: Denis Gradinarov; dateIdentified: 2026; **Event:** samplingProtocol: under bark of *Populus nigra*; verbatimEventDate: 03.xi.2012; habitat: riverine forest
- **Type status:** Other material. **Occurrence:** catalogNumber: BFUS-COL000807; recordedBy: Denis Gradinarov; individualCount: 1; sex: specimen; occurrenceID: 9A30EA4F-B196-5C3A-B3F6-9FCD6584943E; **Location:** country: Bulgaria; stateProvince: Pernik; municipality: Zemen; locality: Zemen Valley; verbatimElevation: 587 m; decimalLatitude: 42.472250; decimalLongitude: 22.737667; geodeticDatum: WGS84; **Identification:** identifiedBy: Denis Gradinarov; dateIdentified: 2026; **Event:** samplingProtocol: under bark of *Populus nigra*; verbatimEventDate: 03.xi.2012; habitat: riverine forest
- **Type status:** Other material. **Occurrence:** catalogNumber: BFUS-COL000808; recordedBy: Denis Gradinarov; individualCount: 1; sex: specimen; occurrenceID: A202072A-0896-5999-9656-4B3046D2602C; **Location:** country: Bulgaria; stateProvince: Pernik; municipality: Zemen; locality: Zemen Valley; verbatimElevation: 587 m; decimalLatitude: 42.472250; decimalLongitude: 22.737667; geodeticDatum: WGS84; **Identification:** identifiedBy: Denis Gradinarov; dateIdentified: 2026; **Event:** samplingProtocol: under bark of *Populus nigra*; verbatimEventDate: 03.xi.2012; habitat: riverine forest
- **Type status:** Other material. **Occurrence:** catalogNumber: BFUS-COL000809; recordedBy: Denis Gradinarov; individualCount: 1; sex: specimen; occurrenceID: 01DA15EE-2DDA-5796-922C-219E8AB47AFA; **Location:** country: Bulgaria; stateProvince: Pernik; municipality: Zemen; locality: Zemen Valley; verbatimElevation: 587 m; decimalLatitude: 42.472250; decimalLongitude: 22.737667; geodeticDatum: WGS84; **Identification:** identifiedBy: Denis Gradinarov; dateIdentified: 2026; **Event:** samplingProtocol: under bark of *Populus nigra*; verbatimEventDate: 03.xi.2012; habitat: riverine forest
- **Type status:** Other material. **Occurrence:** catalogNumber: BFUS-COL000810; recordedBy: Denis Gradinarov; individualCount: 1; sex: specimen; occurrenceID: 9BF7E1DA-4925-5F7A-A84D-10396C514BC2; **Location:** country: Bulgaria; stateProvince: Pernik; municipality: Zemen; locality: Zemen Valley; verbatimElevation: 587 m; decimalLatitude: 42.472250; decimalLongitude: 22.737667; geodeticDatum: WGS84; **Identification:** identifiedBy: Denis Gradinarov; dateIdentified: 2026; **Event:** samplingProtocol: under bark of *Populus nigra*; verbatimEventDate: 03.xi.2012; habitat: riverine forest
- **Type status:** Other material. **Occurrence:** catalogNumber: BFUS-COL000811; recordedBy: Denis Gradinarov; individualCount: 1; sex: specimen; occurrenceID: 770B80CD-6819-5B7D-9029-F59A7E993267; **Location:** country: Bulgaria; stateProvince: Pernik; municipality: Zemen; locality: Zemen Valley; verbatimElevation: 587 m; decimalLatitude: 42.472250; decimalLongitude: 22.737667; geodeticDatum: WGS84; **Identification:** identifiedBy: Denis Gradinarov; dateIdentified: 2026; **Event:** samplingProtocol: under bark of *Populus nigra*; verbatimEventDate: 03.xi.2012; habitat: riverine forest
- **Type status:** Other material. **Occurrence:** catalogNumber: BFUS-COL000812; recordedBy: Denis Gradinarov; individualCount: 1; sex: specimen; occurrenceID: 83A39F11-BB4C-5A13-887B-5827A9B7CB23; **Location:** country: Bulgaria; stateProvince: Pernik; municipality: Zemen; locality: Zemen Valley; verbatimElevation: 587 m; decimalLatitude: 42.472250; decimalLongitude: 22.737667; geodeticDatum: WGS84; **Identification:** identifiedBy: Denis Gradinarov; dateIdentified: 2026; **Event:** samplingProtocol: under bark of *Populus nigra*; verbatimEventDate: 03.xi.2012; habitat: riverine forest
- **Type status:** Other material. **Occurrence:** catalogNumber: BFUS-COL000813; recordedBy: Denis Gradinarov; individualCount: 1; sex: specimen; occurrenceID: 1DF35888-B7C0-5D13-9636-237C04489AED; **Location:** country: Bulgaria; stateProvince: Pernik; municipality: Zemen; locality: Zemen Valley; verbatimElevation: 587 m; decimalLatitude: 42.472250; decimalLongitude: 22.737667; geodeticDatum: WGS84; **Identification:** identifiedBy: Denis Gradinarov; dateIdentified: 2026; **Event:** samplingProtocol: under bark of *Populus nigra*; verbatimEventDate: 03.xi.2012; habitat: riverine forest
- **Type status:** Other material. **Occurrence:** catalogNumber: BFUS-COL000814; recordedBy: Denis Gradinarov; individualCount: 1; sex: specimen; occurrenceID: 0D0417EB-0FB6-52E3-9FD5-8FB7AF64B7B9; **Location:** country: Bulgaria; stateProvince: Pernik; municipality: Zemen; locality: Zemen Valley; verbatimElevation: 587 m; decimalLatitude: 42.472250; decimalLongitude: 22.737667; geodeticDatum: WGS84; **Identification:** identifiedBy: Denis Gradinarov; dateIdentified: 2026; **Event:** samplingProtocol: under bark of *Populus nigra*; verbatimEventDate: 03.xi.2012; habitat: riverine forest
- **Type status:** Other material. **Occurrence:** catalogNumber: BFUS-COL000815; recordedBy: Denis Gradinarov; individualCount: 1; sex: specimen; occurrenceID: 600E91DA-8C9E-5271-837C-6E01615B4535; **Location:** country: Bulgaria; stateProvince: Pernik; municipality: Zemen; locality: Zemen Valley; verbatimElevation: 587 m; decimalLatitude: 42.472250; decimalLongitude: 22.737667; geodeticDatum: WGS84; **Identification:** identifiedBy: Denis Gradinarov; dateIdentified: 2026; **Event:** samplingProtocol: under bark of *Populus nigra*; verbatimEventDate: 03.xi.2012; habitat: riverine forest
- **Type status:** Other material. **Occurrence:** catalogNumber: BFUS-COL000816; recordedBy: Denis Gradinarov; individualCount: 1; sex: specimen; occurrenceID: F172DC6F-5EA4-5F4B-A140-6E362A44DC69; **Location:** country: Bulgaria; stateProvince: Pernik; municipality: Zemen; locality: Zemen Valley; verbatimElevation: 587 m; decimalLatitude: 42.472250; decimalLongitude: 22.737667; geodeticDatum: WGS84; **Identification:** identifiedBy: Denis Gradinarov; dateIdentified: 2026; **Event:** samplingProtocol: under bark of *Populus nigra*; verbatimEventDate: 03.xi.2012; habitat: riverine forest
- **Type status:** Other material. **Occurrence:** catalogNumber: BFUS-COL000817; recordedBy: Denis Gradinarov; individualCount: 1; sex: specimen; occurrenceID: 9A8ECEE4-1408-51A0-9E1C-E9E2506F74A4; **Location:** country: Bulgaria; stateProvince: Pernik; municipality: Zemen; locality: Zemen Valley; verbatimElevation: 587 m; decimalLatitude: 42.472250; decimalLongitude: 22.737667; geodeticDatum: WGS84; **Identification:** identifiedBy: Denis Gradinarov; dateIdentified: 2026; **Event:** samplingProtocol: under bark of *Populus nigra*; verbatimEventDate: 03.xi.2012; habitat: riverine forest
- **Type status:** Other material. **Occurrence:** catalogNumber: BFUS-COL000818; recordedBy: Denis Gradinarov; individualCount: 1; sex: specimen; occurrenceID: B9AF2DEF-1439-546B-B0BC-3CBBF0479D03; **Location:** country: Bulgaria; stateProvince: Pernik; municipality: Zemen; locality: Zemen Valley; verbatimElevation: 587 m; decimalLatitude: 42.472250; decimalLongitude: 22.737667; geodeticDatum: WGS84; **Identification:** identifiedBy: Denis Gradinarov; dateIdentified: 2026; **Event:** samplingProtocol: under bark of *Populus nigra*; verbatimEventDate: 03.xi.2012; habitat: riverine forest
- **Type status:** Other material. **Occurrence:** catalogNumber: BFUS-COL000819; recordedBy: Denis Gradinarov; individualCount: 1; sex: specimen; occurrenceID: 45F01E23-A8FE-5B3B-B358-A1C53A65EBA0; **Location:** country: Bulgaria; stateProvince: Pernik; municipality: Zemen; locality: Zemen Valley; verbatimElevation: 587 m; decimalLatitude: 42.472250; decimalLongitude: 22.737667; geodeticDatum: WGS84; **Identification:** identifiedBy: Denis Gradinarov; dateIdentified: 2026; **Event:** samplingProtocol: under bark of *Populus nigra*; verbatimEventDate: 03.xi.2012; habitat: riverine forest
- **Type status:** Other material. **Occurrence:** catalogNumber: BFUS-COL000820; recordedBy: Denis Gradinarov; individualCount: 1; sex: specimen; occurrenceID: F7C28627-A4DA-581E-AD5D-DECE644CB69B; **Location:** country: Bulgaria; stateProvince: Pernik; municipality: Zemen; locality: Zemen Valley; verbatimElevation: 587 m; decimalLatitude: 42.472250; decimalLongitude: 22.737667; geodeticDatum: WGS84; **Identification:** identifiedBy: Denis Gradinarov; dateIdentified: 2026; **Event:** samplingProtocol: under bark of *Populus nigra*; verbatimEventDate: 03.xi.2012; habitat: riverine forest
- **Type status:** Other material. **Occurrence:** catalogNumber: BFUS-COL000821; recordedBy: Denis Gradinarov; individualCount: 1; sex: specimen; occurrenceID: 6EC438E4-DE35-54BB-9332-E2ADAF3DAC18; **Location:** country: Bulgaria; stateProvince: Pernik; municipality: Zemen; locality: Zemen Valley; verbatimElevation: 587 m; decimalLatitude: 42.472250; decimalLongitude: 22.737667; geodeticDatum: WGS84; **Identification:** identifiedBy: Denis Gradinarov; dateIdentified: 2026; **Event:** samplingProtocol: under bark of *Populus nigra*; verbatimEventDate: 03.xi.2012; habitat: riverine forest
- **Type status:** Other material. **Occurrence:** catalogNumber: BFUS-COL000822; recordedBy: Denis Gradinarov; individualCount: 1; sex: specimen; occurrenceID: CC4F20C4-E9EA-581E-86DA-0C2A30C053C1; **Location:** country: Bulgaria; stateProvince: Pernik; municipality: Zemen; locality: Zemen Valley; verbatimElevation: 587 m; decimalLatitude: 42.472250; decimalLongitude: 22.737667; geodeticDatum: WGS84; **Identification:** identifiedBy: Denis Gradinarov; dateIdentified: 2026; **Event:** samplingProtocol: under bark of *Populus nigra*; verbatimEventDate: 03.xi.2012; habitat: riverine forest
- **Type status:** Other material. **Occurrence:** catalogNumber: BFUS-COL000823; recordedBy: Denis Gradinarov; individualCount: 1; sex: specimen; occurrenceID: 67F63F77-7225-5410-B686-72179C5EFEC5; **Location:** country: Bulgaria; stateProvince: Pernik; municipality: Zemen; locality: Zemen Valley; verbatimElevation: 587 m; decimalLatitude: 42.472250; decimalLongitude: 22.737667; geodeticDatum: WGS84; **Identification:** identifiedBy: Denis Gradinarov; dateIdentified: 2026; **Event:** samplingProtocol: under bark of *Populus nigra*; verbatimEventDate: 03.xi.2012; habitat: riverine forest
- **Type status:** Other material. **Occurrence:** catalogNumber: BFUS-COL000824; recordedBy: Denis Gradinarov; individualCount: 1; sex: specimen; occurrenceID: 5D56D73E-1FCC-5B1F-8E0D-F1D86862338A; **Location:** country: Bulgaria; stateProvince: Pernik; municipality: Zemen; locality: Zemen Valley; verbatimElevation: 587 m; decimalLatitude: 42.472250; decimalLongitude: 22.737667; geodeticDatum: WGS84; **Identification:** identifiedBy: Denis Gradinarov; dateIdentified: 2026; **Event:** samplingProtocol: under bark of *Populus nigra*; verbatimEventDate: 03.xi.2012; habitat: riverine forest
- **Type status:** Other material. **Occurrence:** catalogNumber: BFUS-COL000825; recordedBy: Denis Gradinarov; individualCount: 1; sex: specimen; occurrenceID: C0A113FA-6347-5587-A159-A7BD05C8EE7A; **Location:** country: Bulgaria; stateProvince: Pernik; municipality: Zemen; locality: Zemen Valley; verbatimElevation: 587 m; decimalLatitude: 42.472250; decimalLongitude: 22.737667; geodeticDatum: WGS84; **Identification:** identifiedBy: Denis Gradinarov; dateIdentified: 2026; **Event:** samplingProtocol: under bark of *Populus nigra*; verbatimEventDate: 03.xi.2012; habitat: riverine forest
- **Type status:** Other material. **Occurrence:** catalogNumber: BFUS-COL000826; recordedBy: Denis Gradinarov; individualCount: 1; sex: specimen; occurrenceID: 4A2F647A-658E-523F-B2A0-C901EC752645; **Location:** country: Bulgaria; stateProvince: Pernik; municipality: Zemen; locality: Zemen Valley; verbatimElevation: 587 m; decimalLatitude: 42.472250; decimalLongitude: 22.737667; geodeticDatum: WGS84; **Identification:** identifiedBy: Denis Gradinarov; dateIdentified: 2026; **Event:** samplingProtocol: under bark of *Populus nigra*; verbatimEventDate: 03.xi.2012; habitat: riverine forest
- **Type status:** Other material. **Occurrence:** catalogNumber: BFUS-COL000827; recordedBy: Denis Gradinarov; individualCount: 1; sex: specimen; occurrenceID: C38CB674-27F0-58A1-890D-D71627AF4DF0; **Location:** country: Bulgaria; stateProvince: Pernik; municipality: Zemen; locality: Zemen Valley; verbatimElevation: 587 m; decimalLatitude: 42.472250; decimalLongitude: 22.737667; geodeticDatum: WGS84; **Identification:** identifiedBy: Denis Gradinarov; dateIdentified: 2026; **Event:** samplingProtocol: under bark of *Populus nigra*; verbatimEventDate: 03.xi.2012; habitat: riverine forest
- **Type status:** Other material. **Occurrence:** catalogNumber: BFUS-COL000828; recordedBy: Denis Gradinarov; individualCount: 1; sex: specimen; occurrenceID: CA18FD4E-FEF1-5974-ACAB-4B89D62D6A59; **Location:** country: Bulgaria; stateProvince: Pernik; municipality: Zemen; locality: Zemen Valley; verbatimElevation: 587 m; decimalLatitude: 42.472250; decimalLongitude: 22.737667; geodeticDatum: WGS84; **Identification:** identifiedBy: Denis Gradinarov; dateIdentified: 2026; **Event:** samplingProtocol: under bark of *Populus nigra*; verbatimEventDate: 03.xi.2012; habitat: riverine forest
- **Type status:** Other material. **Occurrence:** catalogNumber: BFUS-COL000829; recordedBy: Denis Gradinarov; individualCount: 1; sex: specimen; occurrenceID: 0CBC0212-880A-53B7-85FF-425C4055E099; **Location:** country: Bulgaria; stateProvince: Pernik; municipality: Zemen; locality: Zemen Valley; verbatimElevation: 587 m; decimalLatitude: 42.472250; decimalLongitude: 22.737667; geodeticDatum: WGS84; **Identification:** identifiedBy: Denis Gradinarov; dateIdentified: 2026; **Event:** samplingProtocol: under bark of *Populus nigra*; verbatimEventDate: 03.xi.2012; habitat: riverine forest
- **Type status:** Other material. **Occurrence:** catalogNumber: BFUS-COL000830; recordedBy: Denis Gradinarov; individualCount: 1; sex: specimen; occurrenceID: A7086FA7-4751-583C-8152-30AD3FBC8F4A; **Location:** country: Bulgaria; stateProvince: Pernik; municipality: Zemen; locality: Zemen Valley; verbatimElevation: 587 m; decimalLatitude: 42.472250; decimalLongitude: 22.737667; geodeticDatum: WGS84; **Identification:** identifiedBy: Denis Gradinarov; dateIdentified: 2026; **Event:** samplingProtocol: under bark of *Populus nigra*; verbatimEventDate: 03.xi.2012; habitat: riverine forest
- **Type status:** Other material. **Occurrence:** catalogNumber: BFUS-COL000831; recordedBy: Denis Gradinarov; individualCount: 1; sex: specimen; occurrenceID: AFD5FF2F-EEFA-5D8D-BAAB-02372F826D43; **Location:** country: Bulgaria; stateProvince: Pernik; municipality: Zemen; locality: Zemen Valley; verbatimElevation: 587 m; decimalLatitude: 42.472250; decimalLongitude: 22.737667; geodeticDatum: WGS84; **Identification:** identifiedBy: Denis Gradinarov; dateIdentified: 2026; **Event:** samplingProtocol: under bark of *Populus nigra*; verbatimEventDate: 03.xi.2012; habitat: riverine forest
- **Type status:** Other material. **Occurrence:** catalogNumber: BFUS-COL000832; recordedBy: Denis Gradinarov; individualCount: 1; sex: specimen; occurrenceID: 1FE9C9B6-EE92-5B00-A5A0-4C96BD9AF2CF; **Location:** country: Bulgaria; stateProvince: Pernik; municipality: Zemen; locality: Zemen Valley; verbatimElevation: 587 m; decimalLatitude: 42.472250; decimalLongitude: 22.737667; geodeticDatum: WGS84; **Identification:** identifiedBy: Denis Gradinarov; dateIdentified: 2026; **Event:** samplingProtocol: under bark of *Populus nigra*; verbatimEventDate: 03.xi.2012; habitat: riverine forest
- **Type status:** Other material. **Occurrence:** catalogNumber: BFUS-COL000833; recordedBy: Denis Gradinarov; individualCount: 1; sex: specimen; occurrenceID: 1006C246-64CE-5AED-93C9-302F72ED8D07; **Location:** country: Bulgaria; stateProvince: Pernik; municipality: Zemen; locality: Zemen Valley; verbatimElevation: 587 m; decimalLatitude: 42.472250; decimalLongitude: 22.737667; geodeticDatum: WGS84; **Identification:** identifiedBy: Denis Gradinarov; dateIdentified: 2026; **Event:** samplingProtocol: under bark of *Populus nigra*; verbatimEventDate: 03.xi.2012; habitat: riverine forest
- **Type status:** Other material. **Occurrence:** catalogNumber: BFUS-COL000834; recordedBy: Ognyan Sivilov; individualCount: 1; sex: female; occurrenceID: C2BDED6A-4E3B-5A1C-9A47-EEB2371D40E1; **Location:** country: Bulgaria; stateProvince: Blagoevgrad; municipality: Petrich; locality: Sandanski-Petrich Valley, SE of Starchevo Village; verbatimElevation: 98 m; decimalLatitude: 41.467211; decimalLongitude: 23.268465; geodeticDatum: WGS84; **Identification:** identifiedBy: Denis Gradinarov; dateIdentified: 2026; **Event:** samplingProtocol: under bark of *Populus alba*; verbatimEventDate: 29.v.2013; habitat: riverine forest
- **Type status:** Other material. **Occurrence:** catalogNumber: BFUS-COL000835; recordedBy: Ognyan Sivilov; individualCount: 1; sex: specimen; occurrenceID: F68BFB36-6A1A-55F0-9F8C-B998CF233897; **Location:** country: Bulgaria; stateProvince: Blagoevgrad; municipality: Sandanski; locality: Pirin Mountains, NE of Kalimantsi Village; verbatimElevation: 300 m; decimalLatitude: 41.46451; decimalLongitude: 23.49091; geodeticDatum: WGS84; **Identification:** identifiedBy: Denis Gradinarov; dateIdentified: 2026; **Event:** samplingProtocol: under alder bark; verbatimEventDate: 28.vi.2019; habitat: riverine forest
- **Type status:** Other material. **Occurrence:** catalogNumber: BFUS-COL000836; recordedBy: Ognyan Sivilov; individualCount: 1; sex: specimen; occurrenceID: 9F666FC3-7089-5513-9062-944B78F8B31A; **Location:** country: Bulgaria; stateProvince: Pleven; municipality: Gulyantsi; locality: Western Danubian Plain, N of Gulyantsi; verbatimElevation: 25 m; decimalLatitude: 43.68686598; decimalLongitude: 24.70856; geodeticDatum: WGS84; **Identification:** identifiedBy: Denis Gradinarov; dateIdentified: 2026; **Event:** samplingProtocol: under bark of *Populus* sp.; verbatimEventDate: 18.v.2021; habitat: clearing area
- **Type status:** Other material. **Occurrence:** catalogNumber: BFUS-COL000837; recordedBy: Ognyan Sivilov; individualCount: 1; sex: specimen; occurrenceID: 62FBE2E2-85A3-5D6E-AA06-AB44E8FBF326; **Location:** country: Bulgaria; stateProvince: Pleven; municipality: Gulyantsi; locality: Western Danubian Plain, N of Gulyantsi; verbatimElevation: 25 m; decimalLatitude: 43.68686598; decimalLongitude: 24.70856; geodeticDatum: WGS84; **Identification:** identifiedBy: Denis Gradinarov; dateIdentified: 2026; **Event:** samplingProtocol: under bark of *Populus* sp.; verbatimEventDate: 18.v.2021; habitat: clearing area
- **Type status:** Other material. **Occurrence:** catalogNumber: BFUS-COL000838; recordedBy: Ognyan Sivilov; individualCount: 1; sex: specimen; occurrenceID: C2444C13-73E1-5135-9562-FB72DEF9D60C; **Location:** country: Bulgaria; stateProvince: Pleven; municipality: Dolna Mitropolia; locality: Middle Danubian Plain, N of Riben Village; verbatimElevation: 41 m; decimalLatitude: 43.54513202; decimalLongitude: 24.61941; geodeticDatum: WGS84; **Identification:** identifiedBy: Denis Gradinarov; dateIdentified: 2026; **Event:** samplingProtocol: under bark of *Populus* sp.; verbatimEventDate: 19.v.2021; habitat: riverine vegetation
- **Type status:** Other material. **Occurrence:** catalogNumber: BFUS-COL000839; recordedBy: Ognyan Sivilov; individualCount: 1; sex: specimen; occurrenceID: B3B420F6-546C-5723-A3D4-7EF99126AA37; **Location:** country: Bulgaria; stateProvince: Silistra; municipality: Glavinitsa; locality: Eastern Danubian Plain, N of Dolno Ryahovo Village; verbatimElevation: 15 m; decimalLatitude: 44.08248402; decimalLongitude: 26.78935; geodeticDatum: WGS84; **Identification:** identifiedBy: Denis Gradinarov; dateIdentified: 2026; **Event:** samplingProtocol: under bark of *Salix* sp.; verbatimEventDate: 07.vii.2021; habitat: riverine vegetation
- **Type status:** Other material. **Occurrence:** catalogNumber: BFUS-COL000840; recordedBy: Ognyan Sivilov; individualCount: 1; sex: specimen; occurrenceID: 152A0296-A02D-5CC2-86F6-C8FCDF497E64; **Location:** country: Bulgaria; stateProvince: Silistra; municipality: Glavinitsa; locality: Eastern Danubian Plain, N of Dolno Ryahovo Village; verbatimElevation: 15 m; decimalLatitude: 44.08248402; decimalLongitude: 26.78935; geodeticDatum: WGS84; **Identification:** identifiedBy: Denis Gradinarov; dateIdentified: 2026; **Event:** samplingProtocol: under bark of *Salix* sp.; verbatimEventDate: 07.vii.2021; habitat: riverine vegetation
- **Type status:** Other material. **Occurrence:** catalogNumber: BFUS-COL000841; recordedBy: Ognyan Sivilov; individualCount: 1; sex: specimen; occurrenceID: 5AF446EC-8903-55E9-8D7B-7C1A62B3E22F; **Location:** country: Bulgaria; stateProvince: Ruse; municipality: Borovo; locality: Eastern Danubian Plain, N of Batin Village; verbatimElevation: 18 m; decimalLatitude: 43.67351001; decimalLongitude: 25.6888059; geodeticDatum: WGS84; **Identification:** identifiedBy: Denis Gradinarov; dateIdentified: 2026; **Event:** samplingProtocol: under bark of *Salix* sp.; verbatimEventDate: 07.vii.2021; habitat: riverine vegetation
- **Type status:** Other material. **Occurrence:** catalogNumber: BFUS-COL000842; recordedBy: Ognyan Sivilov; individualCount: 1; sex: specimen; occurrenceID: 77D90DAE-37D4-56A9-9D9A-C764F8A1D056; **Location:** country: Bulgaria; stateProvince: Vratsa; municipality: Oryahovo; locality: Western Danubian Plain, N of Galovo Village; verbatimElevation: 43 m; decimalLatitude: 43.66895101; decimalLongitude: 24.07607599; geodeticDatum: WGS84; **Identification:** identifiedBy: Denis Gradinarov; dateIdentified: 2026; **Event:** samplingProtocol: on trunk of living *Populus alba*; verbatimEventDate: 08.vii.2021; habitat: deciduous forest
- **Type status:** Other material. **Occurrence:** catalogNumber: BFUS-COL000843; recordedBy: Ognyan Sivilov; individualCount: 1; sex: specimen; occurrenceID: 644930BF-DEA9-50DD-B7A2-2FD1BA7546A0; **Location:** country: Bulgaria; stateProvince: Vratsa; municipality: Oryahovo; locality: Western Danubian Plain, N of Galovo Village; verbatimElevation: 43 m; decimalLatitude: 43.66895101; decimalLongitude: 24.07607599; geodeticDatum: WGS84; **Identification:** identifiedBy: Denis Gradinarov; dateIdentified: 2026; **Event:** samplingProtocol: on trunk of living *Populus alba*; verbatimEventDate: 08.vii.2021; habitat: deciduous forest
- **Type status:** Other material. **Occurrence:** catalogNumber: BFUS-COL000844; recordedBy: Denis Gradinarov; individualCount: 1; sex: specimen; occurrenceID: 123836BD-FC3A-5BE2-A93B-FA982822A9BC; **Location:** country: Bulgaria; stateProvince: Blagoevgrad; municipality: Simitli; locality: Maleshevska Planina Mountains, SW of Polena Village; verbatimElevation: 461 m; decimalLatitude: 41.833917; decimalLongitude: 23.094767; geodeticDatum: WGS84; **Identification:** identifiedBy: Denis Gradinarov; dateIdentified: 2026; **Event:** samplingProtocol: at flight; verbatimEventDate: 25.iv.2022; habitat: roadside vegetation
- **Type status:** Other material. **Occurrence:** catalogNumber: BFUS-COL000845; recordedBy: Denis Gradinarov; individualCount: 1; sex: specimen; occurrenceID: 458B6F85-246E-5E13-9CF8-71328D2E3202; **Location:** country: Bulgaria; stateProvince: Sofia City; municipality: Stolichna; locality: Lozenska Planina Mountains, N of Dolni Pasarel Village; verbatimElevation: 886 m; decimalLatitude: 42.567250; decimalLongitude: 23.499283; geodeticDatum: WGS84; **Identification:** identifiedBy: Denis Gradinarov; dateIdentified: 2026; **Event:** samplingProtocol: under bark; verbatimEventDate: 07.v.2023; habitat: oak forest
- **Type status:** Other material. **Occurrence:** catalogNumber: BFUS-COL000846; recordedBy: Denis Gradinarov; individualCount: 1; sex: specimen; occurrenceID: E4D99C03-47E4-5FF1-9A53-3C148F9E7312; **Location:** country: Bulgaria; stateProvince: Sofia City; municipality: Stolichna; locality: Lozenska Planina Mountains, N of Dolni Pasarel Village; verbatimElevation: 886 m; decimalLatitude: 42.567250; decimalLongitude: 23.499283; geodeticDatum: WGS84; **Identification:** identifiedBy: Denis Gradinarov; dateIdentified: 2026; **Event:** samplingProtocol: under bark; verbatimEventDate: 07.v.2023; habitat: oak forest

##### Distribution

Azerbaijan, Albania, Armenia, Austria, Belgium, Bosnia and Herzegovina, Bulgaria, Belarus, Croatia, Czechia, Denmark, Estonia, Finland, France, Great Britain, Germany, Georgia, Greece, Hungary, Italy, Latvia, Liechtenstein, Kazakhstan, Malta, North Macedonia, Moldova, The Netherlands, Norway, Poland, Romania, Russia (South, Central and North European Territory, East and West Siberia, Far East), Slovakia, Slovenia, Spain, Sweden, Switzerland, Ukraine, Serbia and Montenegro, Kazakhstan, Mongolia, Algeria, Tunisia, Nearctic Region (Schuh 2020); Iran (Otero and Ghahari 2020, Farashiani et al. 2022).

##### Notes

**Figure 8.**
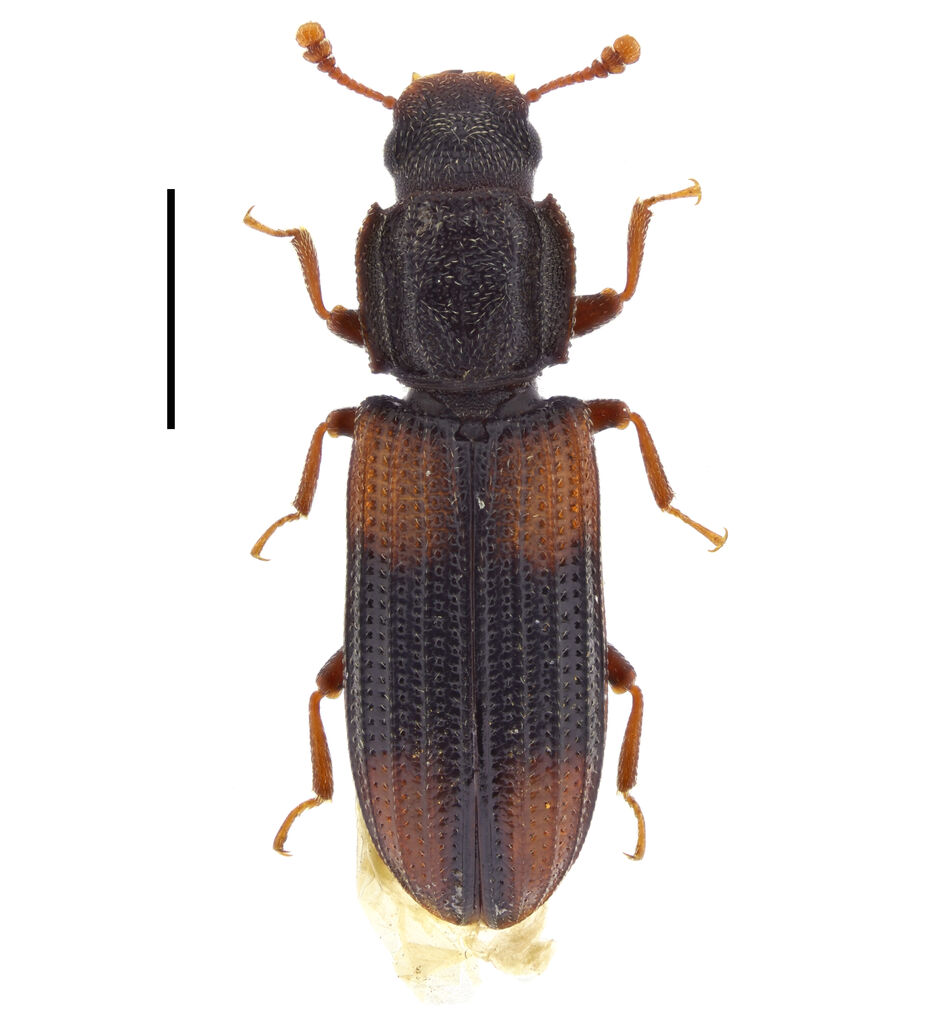
*Bitoma crenata* (Fabricius, 1775) from western Danubian Plain, Bulgaria (BFUS-COL000836). Scale bar: 1 mm.

In Bulgaria, this species has been recorded from Sarnena Sredna Gora Mountains (Joakimov (1904): 16, as *Ditoma crenata*) and from Maleshevska Planina Mountains (Guéorguiev and Ljubomirov 2009: 254). Here, we record *B. crenata* from a number of localities from Danubian Plain (Fig. 8) and south-western Bulgaria.

#### Colobicus

Latreille, 1807

6FB05B20-2436-5DF0-801B-4D45A832ACA6

#### Colobicus hirtus

(Rossi, 1790)

FA5BBAE4-00B1-5172-B4AB-01EF2D02336D

##### Materials

- **Type status:** Other material. **Occurrence:** catalogNumber: BFUS-COL000847; recordedBy: Ognyan Sivilov & Boyan Zlatkov; individualCount: 1; sex: male; occurrenceID: D81E3163-0D57-5463-8170-D40FD6828FC5; **Location:** country: Bulgaria; stateProvince: Blagoevgrad; municipality: Hadzhidimovo; locality: SE of Paril Village; verbatimElevation: 755 m; decimalLatitude: 41.432667; decimalLongitude: 23.700117; geodeticDatum: WGS84; **Identification:** identifiedBy: Denis Gradinarov; dateIdentified: 2026; **Event:** samplingProtocol: at light; verbatimEventDate: 17.vi.2013–18.vi.2013; habitat: riverine vegetation
- **Type status:** Other material. **Occurrence:** catalogNumber: BFUS-COL000848; recordedBy: Denis Gradinarov; individualCount: 1; sex: specimen; occurrenceID: B1A9649D-6CE5-5D33-B989-435F74B2870B; **Location:** country: Bulgaria; stateProvince: Sofia City; municipality: Stolichna; locality: Sofia City, Borisova Gradina Park; verbatimElevation: 615 m; decimalLatitude: 42.678067; decimalLongitude: 23.340433; geodeticDatum: WGS84; **Identification:** identifiedBy: Denis Gradinarov; dateIdentified: 2026; **Event:** samplingProtocol: under bark of *Quercus rubra*; verbatimEventDate: 21.iii.2023; habitat: mixed forest

##### Distribution

Austria, Bosnia and Herzegovina, Bulgaria, Croatia, Czechia, France, Great Britain, Germany, Greece, Hungary, Italy, Liechtenstein, Poland, Russia (South, Central and North European Territory, West Siberia, Far East), Slovakia, Slovenia, Spain, Sweden, Switzerland, Ukraine, Serbia and Montenegro, Afghanistan, Japan, China (Northern Territory), Asian Türkiye, Morocco (Schuh 2020); Romania (Merkl et al. 2016); Iran (Otero and Ghahari 2020, Farashiani et al. 2022); Moldova (Bacal 2023, Bacal and Buşmachiu 2025); Kazakhstan (Temreshev 2025); Belgium (Thomaes et al. 2025).

##### Notes

**Figure 9.**
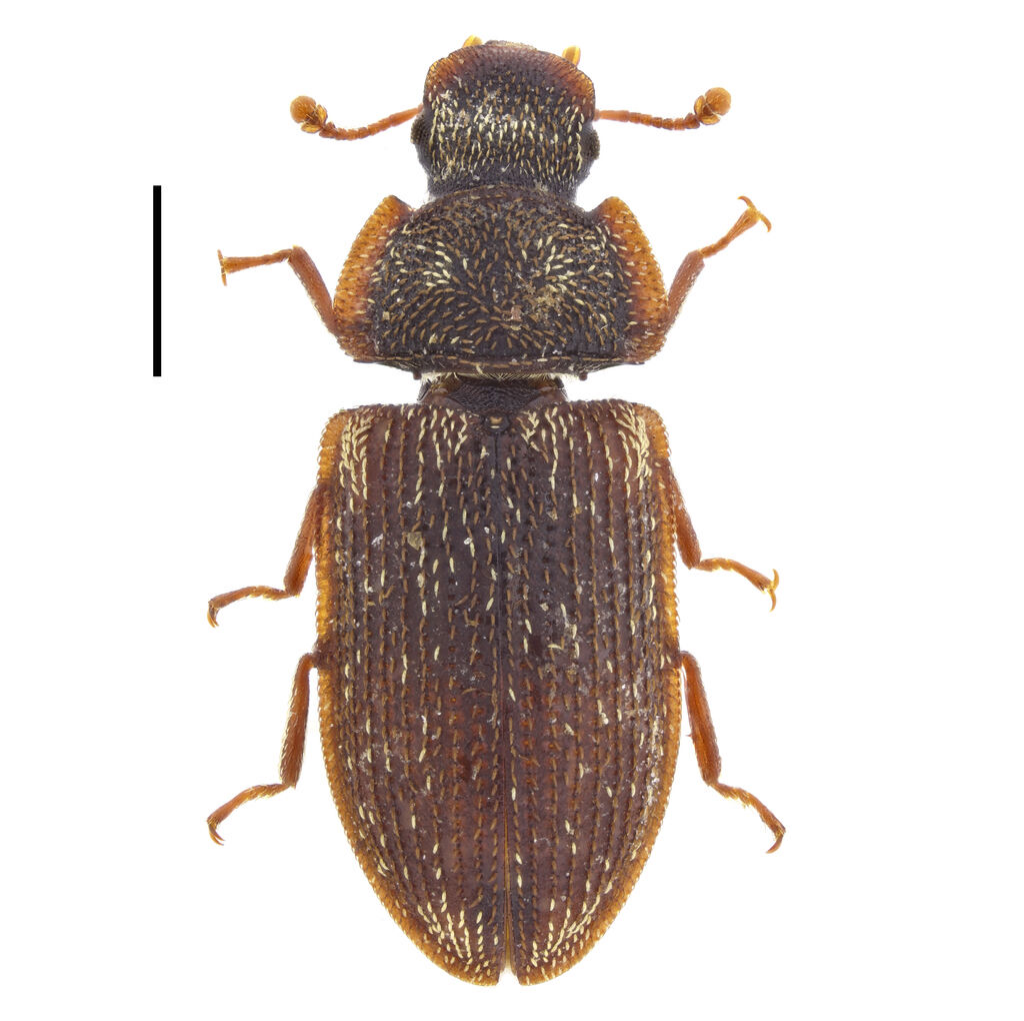
*Colobicus hirtus* (Rossi, 1790) from Paril Village, Bulgaria (male, BFUS-COL000847). Scale bar: 1 mm.

In Bulgaria, this species has been recorded from Varna (Anonymous (1907): 300, as *Colobicus emarginatus*), Emine Cape (Palm 1966: 14), from the vicinity of Ropotamo River mouth (Black Sea coast) (Dajoz 1971: 101), from Maleshevska planina Mountains (Bencheva and Doychev 2022) and from southern Dobrudzha (Trigortsi and Dropla Villages) (Guéorguiev et al. 2026: 4). In the present study, we report *C. hirtus* from the vicinity of Paril Village (between Pirin and Slavyanka Mountains) (Fig. 9) and from Sofia City.

#### Endophloeus

Dejean, 1834

79BE688C-5FC2-574C-A309-731477D468E1

#### Endophloeus markovichianus

(Piller & Mitterpacher, 1783)

038636C1-D0B8-5551-BDEE-34692E0AA8BF

##### Distribution

Austria, Bosnia and Herzegovina, Bulgaria, Croatia, France, Great Britain, Greece, Hungary, Italy, North Macedonia, Poland, Portugal, Romania, Russia, Slovakia, Slovenia, Spain, Ukraine, Serbia and Montenegro, European Türkiye, Algeria, Morocco, Tunisia (Schuh 2020); Iran (Otero and Ghahari 2020); Belgium (Thomaes et al. 2025).

##### Notes

In Bulgaria, this species is known from Emine Cape (Palm 1966: 13), Borovets (Rila Mountains), Petrohan and from the vicinity of Kamchia River mouth (Black Sea coast) (Dajoz 1971: 99).

#### Endophloeus squarrosus

Germar, 1847

4C65AF97-97DB-5186-9260-62004774CE54

##### Distribution

Greece, Serbia and Montenegro, European Türkiye (Schuh 2020); Bulgaria (Roubal 1931).

##### Notes

In Bulgaria, this species has been recorded from Rila Mountains (Roubal 1931: 453). The species is omitted for the country in both editions of the Catalogue of Palaearctic Coleoptera (Ślipiński and Schuh 2008, Schuh 2020).

#### Langelandia

Aubé, 1842

ABC637D4-1051-54D6-884C-BA85B0191C0A

#### Langelandia

Aubé, 1842

B4B35F4A-1E89-5EA0-B6E9-69EE1DAF354C

#### Langelandia (Langelandia) anophthalma

Aubé, 1842

CBAC3DD6-ED7B-562B-8F2A-836248217366

##### Materials

- **Type status:** Other material. **Occurrence:** catalogNumber: BFUS-COL000849; recordedBy: Vladimir Stefanov & Denis Gradinarov; individualCount: 1; sex: female; occurrenceID: 003A78CA-906A-5126-BCF5-659E9D152C7C; **Location:** country: Bulgaria; stateProvince: Sofia City; municipality: Stolichna; locality: Sofia Valley, Kremikovtsi locality; verbatimElevation: 696 m; decimalLatitude: 42.792450; decimalLongitude: 23.492717; geodeticDatum: WGS84; **Identification:** identifiedBy: Denis Gradinarov; dateIdentified: 2026; **Event:** samplingProtocol: under nest of *Spermophilus citellus* L.; verbatimEventDate: 17.vii.2019; habitat: pasture
- **Type status:** Other material. **Occurrence:** catalogNumber: BFUS-COL000850; recordedBy: Vladimir Stefanov & Denis Gradinarov; individualCount: 1; sex: male; occurrenceID: 1ACD2470-950D-5A54-95C8-5A1ED3F1F143; **Location:** country: Bulgaria; stateProvince: Sofia City; municipality: Stolichna; locality: Sofia Valley, Kremikovtsi locality; verbatimElevation: 690 m; decimalLatitude: 42.791567; decimalLongitude: 23.503700; geodeticDatum: WGS84; **Identification:** identifiedBy: Denis Gradinarov; dateIdentified: 2026; **Event:** samplingProtocol: in nest of *Spermophilus citellus* L.; verbatimEventDate: 02.iii.2021; habitat: pasture
- **Type status:** Other material. **Occurrence:** catalogNumber: BFUS-COL000851; recordedBy: Vladimir Stefanov & Denis Gradinarov; individualCount: 1; sex: male; occurrenceID: 38470FDF-8C1A-5968-A9CE-4C6ACCDD8002; **Location:** country: Bulgaria; stateProvince: Sofia City; municipality: Stolichna; locality: Sofia Valley, Kremikovtsi locality; verbatimElevation: 699 m; decimalLatitude: 42.792633; decimalLongitude: 23.491717; geodeticDatum: WGS84; **Identification:** identifiedBy: Denis Gradinarov; dateIdentified: 2026; **Event:** samplingProtocol: in burrow of *Spermophilus citellus* L.; verbatimEventDate: 02.iii.2021; habitat: pasture

##### Distribution

Austria, Czechia, Belgium, Bosnia and Herzegovina, France, Great Britain, Germany, Greece, Hungary, Italy, The Netherlands, Poland, Portugal, Romania, Slovakia, Spain, Switzerland, Ukraine, Serbia and Montenegro (Schuh 2020); Bulgaria (Bekchiev and Guéorguiev 2015; present study).

##### Notes

**Figure 10.**
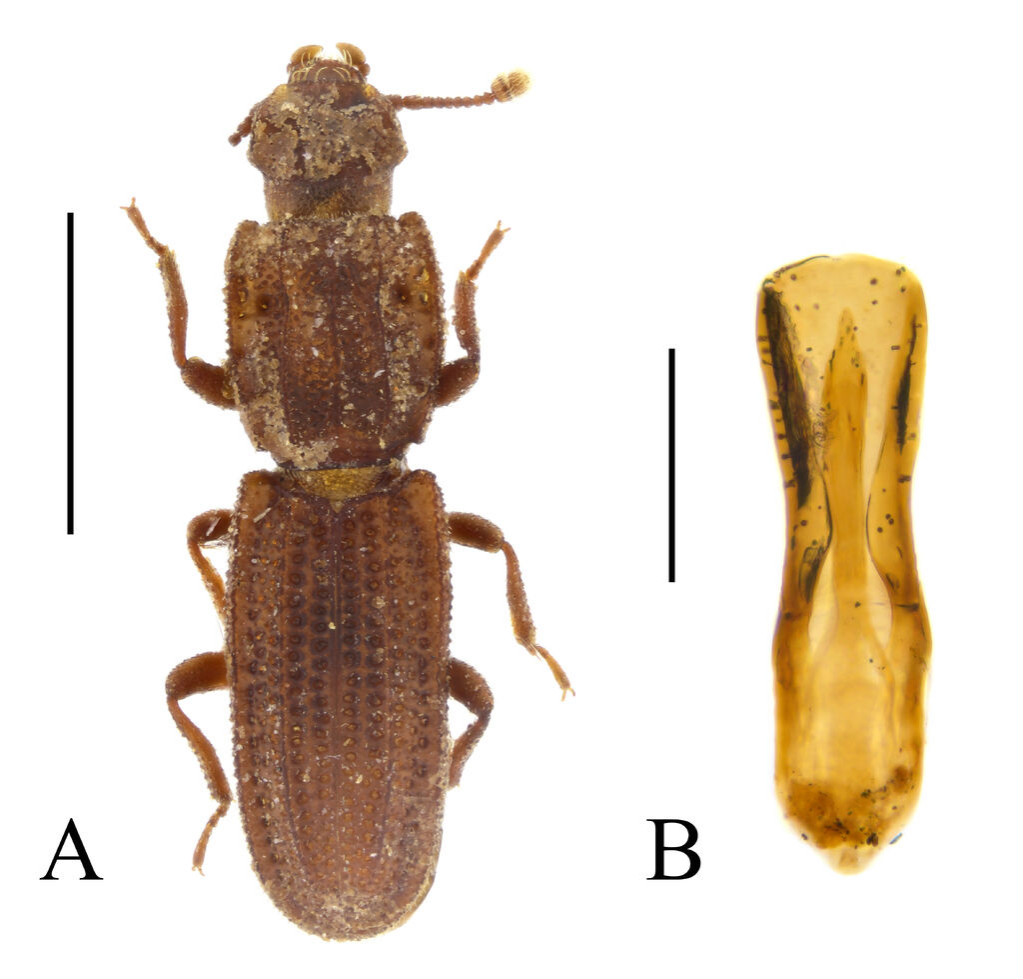
*Langelandia anophthalma* Aubé, 1842 from Sofia Valley, Bulgaria: **A** male (BFUS-COL000850); **B** aedeagus of the same specimen. Scale bars: 1 mm (A); 0.1 mm (B).

*Langelandia anophthalma* is a highly variable species with a wide distributional range in Europe (Dajoz 1969; Orousset 1989). We consider its relatively larger body size and the morphology of the aedeagus as the main features for distinguishing it from other similar species in the subgenus Langelandia (Dajoz 1969; Orousset 1989). For Bulgaria, *L. anophthalma* is mentioned without exact locality by Bekchiev and Guéorguiev (2015): 339. The species is not listed for Bulgaria in both editions of the Catalogue of Palaearctic Coleoptera (Ślipiński and Schuh 2008; Schuh 2020). Herein, we confirm the presence of *L. anophthalma* in Bulgaria and record the notable cases of the species inhabiting the burrows (including the nesting materials) of the European souslik (*Spermophilus citellus* L.) (Fig. 10).

#### Nosodomodes

Reitter, 1922

B4C85373-60FF-59BA-AC2D-947514D70AD3

#### Nosodomodes diabolicus

(Schaufuss, 1862)

6268B837-0B8E-5B00-97E1-3349B9A7AAFB

##### Materials

- **Type status:** Other material. **Occurrence:** catalogNumber: BFUS-COL000852; recordedBy: Yana Petrova; individualCount: 1; sex: male; occurrenceID: 9CA8EE95-B2EA-5224-9612-5ED824C1F009; **Location:** country: Bulgaria; stateProvince: Stara Zagora; municipality: Bratya Daskalovi; locality: Sarnena Gora Mountains, NW of Gorno Novo Selo Village; verbatimElevation: 800 m; decimalLatitude: 42.47418; decimalLongitude: 25.22207; geodeticDatum: WGS84; **Identification:** identifiedBy: Denis Gradinarov; dateIdentified: 2021; **Event:** samplingProtocol: under oak bark; verbatimEventDate: 24.ix.2018; habitat: deciduous forest
- **Type status:** Other material. **Occurrence:** catalogNumber: BFUS-COL000853; recordedBy: Ognyan Sivilov; individualCount: 1; sex: male; occurrenceID: 33A5A1C8-C902-5D5C-B92E-7070BAFF8C80; **Location:** country: Bulgaria; stateProvince: Shumen; municipality: Shumen; locality: Shumensko Plato Nature Park; verbatimElevation: 485 m; decimalLatitude: 43.255567; decimalLongitude: 26.887117; geodeticDatum: WGS84; **Identification:** identifiedBy: Denis Gradinarov; dateIdentified: 2026; **Event:** samplingProtocol: under beech bark; verbatimEventDate: 01.vi.2019; habitat: beech forest
- **Type status:** Other material. **Occurrence:** catalogNumber: BFUS-COL000854; recordedBy: Ognyan Sivilov; individualCount: 1; sex: female; occurrenceID: FD760EFC-5DAE-5A40-B513-F5C659705480; **Location:** country: Bulgaria; stateProvince: Shumen; municipality: Shumen; locality: Shumensko Plato Nature Park; verbatimElevation: 485 m; decimalLatitude: 43.255567; decimalLongitude: 26.887117; geodeticDatum: WGS84; **Identification:** identifiedBy: Denis Gradinarov; dateIdentified: 2026; **Event:** samplingProtocol: under beech bark; verbatimEventDate: 01.vi.2019; habitat: beech forest
- **Type status:** Other material. **Occurrence:** catalogNumber: BFUS-COL000855; recordedBy: Ognyan Sivilov; individualCount: 1; sex: male; occurrenceID: 626D990B-2CB6-50C4-B1FD-E7692F0CC7BD; **Location:** country: Bulgaria; stateProvince: Shumen; municipality: Shumen; locality: Shumensko Plato Nature Park; verbatimElevation: 485 m; decimalLatitude: 43.254167; decimalLongitude: 26.888433; geodeticDatum: WGS84; **Identification:** identifiedBy: Denis Gradinarov; dateIdentified: 2026; **Event:** samplingProtocol: under beech bark; verbatimEventDate: 01.vi.2019; habitat: beech forest

##### Distribution

Armenia, Bulgaria, Greece, North Macedonia, Romania, Ukraine, Serbia and Montenegro, Asian Türkiye (Schuh 2020); Iran (Otero and Ghahari 2020); Moldova (Bacal 2023, Bacal and Buşmachiu 2025).

##### Notes

**Figure 11.**
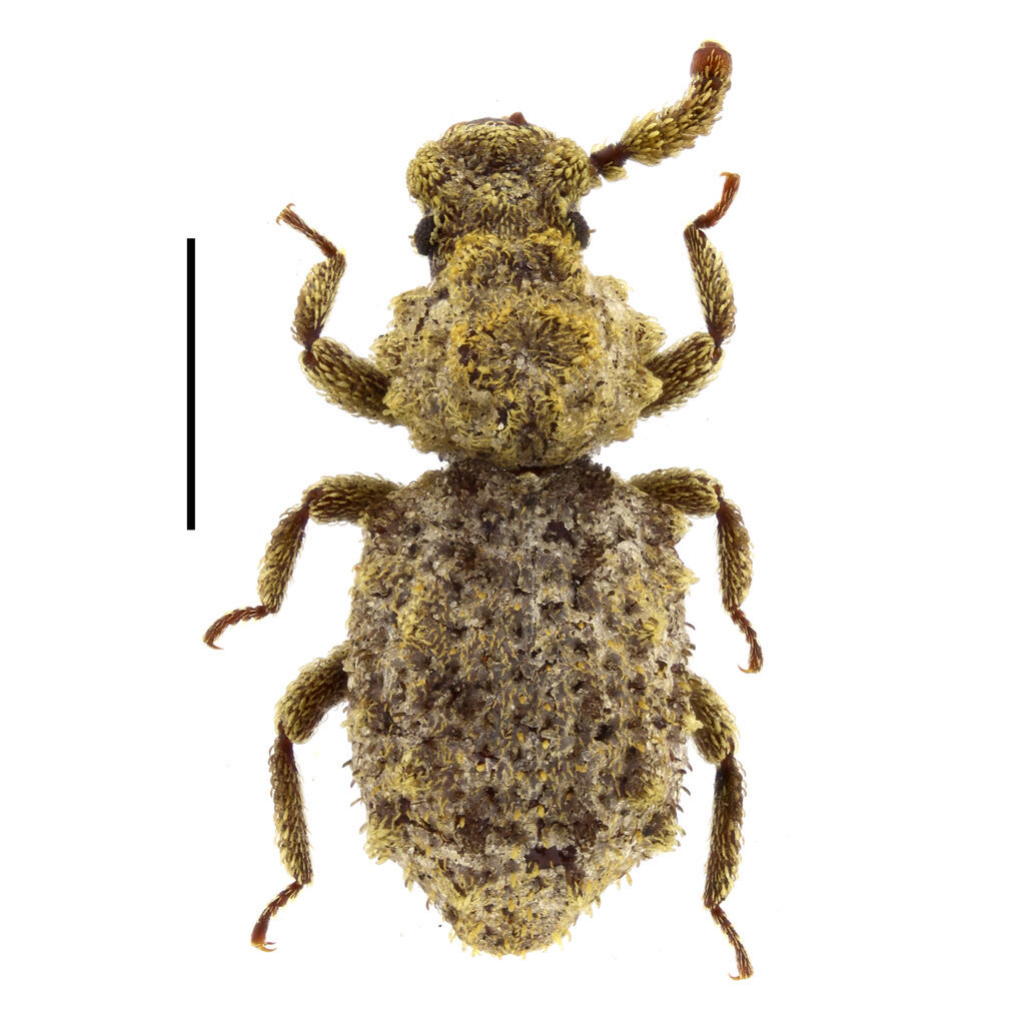
*Nosodomodes diabolicus* (Schaufuss, 1862) from Sarnena Gora Mountains, Bulgaria (male, BFUS-COL000852). Scale bar: 1 mm.

In Bulgaria, this species has been recorded from Emine Cape (Palm (1966): 14, as *Corticus diabolicus*) and from Petrohan (Dajoz (1971): 99, as *Corticus diabolicus*). Here, we record *N. diabolicus* from Sarnena Gora Mountains (Fig. 11) and from Shumensko Plato Nature Park.

#### Nosodomodes tuberculatus

(Germar, 1832)

AC183A92-4024-5211-83D2-A40345D9C99E

##### Materials

- **Type status:** Other material. **Occurrence:** catalogNumber: BFUS-COL000856; recordedBy: Ognyan Sivilov; individualCount: 1; sex: specimen; occurrenceID: 1F942689-81BC-56A4-8DDF-D4BBA3327E27; **Location:** country: Bulgaria; stateProvince: Blagoevgrad; municipality: Gotse Delchev; locality: Pirin Mountains, Popovi Livadi Village; verbatimElevation: 1430 m; decimalLatitude: 41.552674; decimalLongitude: 23.638117; geodeticDatum: WGS84; **Identification:** identifiedBy: Denis Gradinarov; dateIdentified: 2026; **Event:** samplingProtocol: pitfall traps; verbatimEventDate: 18.vi.2012–20.vii.2012; habitat: pine forest
- **Type status:** Other material. **Occurrence:** catalogNumber: BFUS-COL000857; recordedBy: Ognyan Sivilov; individualCount: 1; sex: female; occurrenceID: BBC3A292-F373-5D99-BFAE-CA9B6693570C; **Location:** country: Bulgaria; stateProvince: Blagoevgrad; municipality: Gotse Delchev; locality: Pirin Mountains, Popovi Livadi Village; verbatimElevation: 1430 m; decimalLatitude: 41.552674; decimalLongitude: 23.638117; geodeticDatum: WGS84; **Identification:** identifiedBy: Denis Gradinarov; dateIdentified: 2026; **Event:** samplingProtocol: pitfall traps; verbatimEventDate: 10.ix.2012–17.x.2012; habitat: pine forest

##### Distribution

Bulgaria, Greece, North Macedonia, Romania, South European Territory of Russia, Ukraine (Schuh 2020).

##### Notes

**Figure 12.**
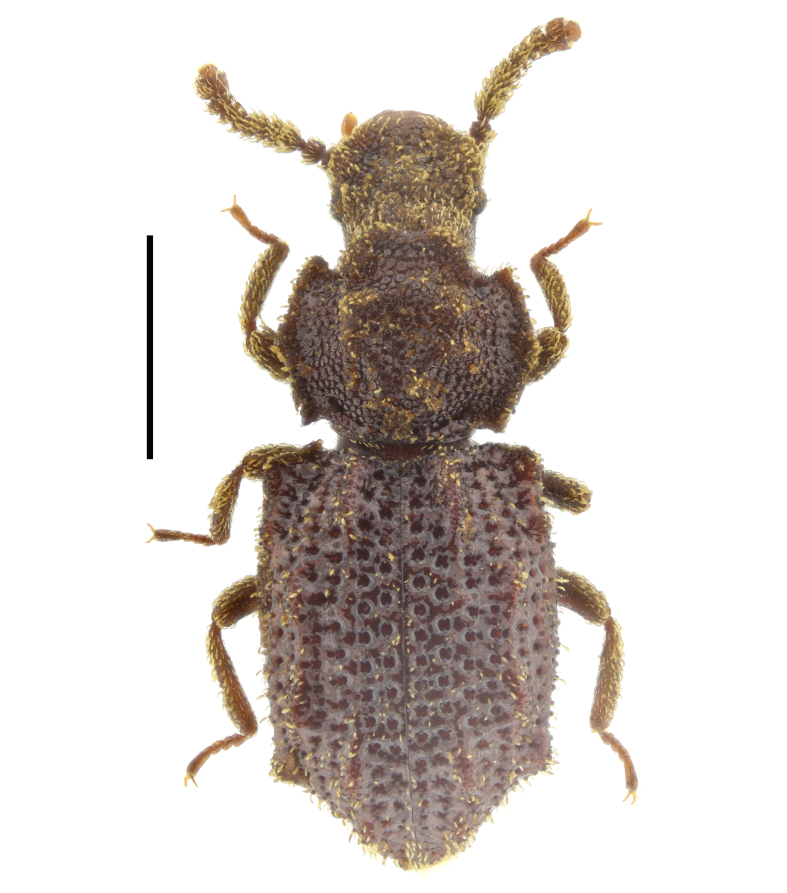
*Nosodomodes tuberculatus* (Germar, 1832) from Pirin Mountains, Bulgaria (female, BFUS-COL000857). Scale bar: 1 mm.

In Bulgaria, this species has been recorded from Sarnena Sredna Gora Mountains (Joakimov (1904): 16, as *Corticus tuberculatus*), Central Stara Planina Mountains (Kozya Stena Hut) (Angelov (1968b): 144, as *Corticus tuberculatus*) and from Belasitsa Mountains (Guéorguiev 2011: 13). Here, we record *N. tuberculatus* from Pirin Mountains (Fig. 12).

#### Synchita

Hellwig, 1792

19416BD8-601C-5D60-BE66-184E93C935A5

#### Synchita humeralis

(Fabricius, 1792)

CD0EA9C8-4EC6-581E-939F-025E5AB38D5E

##### Distribution

Austria, Belgium, Bosnia and Herzegovina, Bulgaria, Croatia, Czechia, Denmark, Finland, France, Great Britain, Germany, Hungary, Italy, Latvia, North Macedonia, Moldova, Norway, Poland, Romania, Russia (South, Central and North European Territory, East Siberia, Far East), Slovakia, Slovenia, Spain, Sweden, Switzerland, Ukraine, Serbia and Montenegro (Schuh 2020).

##### Notes

In Bulgaria, this species has been recorded without exact localities (Ślipiński and Schuh 2008: 84; Schuh 2020: 75).

#### Synchita mediolanensis

A. Villa & J. B. Villa, 1833

008FA0FC-BB3A-5074-B07D-0EE4D125E3FC

##### Materials

- **Type status:** Other material. **Occurrence:** catalogNumber: BFUS-COL000858; recordedBy: Ognyan Sivilov & Boyan Zlatkov; individualCount: 1; sex: specimen; occurrenceID: 4D526E38-B5C2-5339-9324-4BA6687C830A; **Location:** country: Bulgaria; stateProvince: Blagoevgrad; municipality: Petrich; locality: Belasitsa Mountains, above Samuilovo Village; verbatimElevation: 385 m; decimalLatitude: 41.367400; decimalLongitude: 23.091700; geodeticDatum: WGS84; **Identification:** identifiedBy: Denis Gradinarov; dateIdentified: 2026; **Event:** samplingProtocol: at light; verbatimEventDate: 19.vi.2013–20.vi.2013
- **Type status:** Other material. **Occurrence:** catalogNumber: BFUS-COL000859; recordedBy: Ognyan Sivilov & Boyan Zlatkov; individualCount: 1; sex: male; occurrenceID: 5A29A062-0212-5C76-BC70-A53CB7F12326; **Location:** country: Bulgaria; stateProvince: Blagoevgrad; municipality: Petrich; locality: Sandanski-Petrich Valley, SE of Starchevo Village; verbatimElevation: 95 m; decimalLatitude: 41.46812; decimalLongitude: 23.26900; geodeticDatum: WGS84; **Identification:** identifiedBy: Denis Gradinarov; dateIdentified: 2026; **Event:** samplingProtocol: at light; verbatimEventDate: 20.vi.2013–21.vi.2013; habitat: riverine forest
- **Type status:** Other material. **Occurrence:** catalogNumber: BFUS-COL000860; recordedBy: Ognyan Sivilov & Boyan Zlatkov; individualCount: 1; sex: male; occurrenceID: 9775951C-6EF0-50F7-9662-1519588AAE80; **Location:** country: Bulgaria; stateProvince: Blagoevgrad; municipality: Sandanski; locality: Pirin Mountains, NE of Kalimantsi Village; verbatimElevation: 315 m; decimalLatitude: 41.465033; decimalLongitude: 23.488750; geodeticDatum: WGS84; **Identification:** identifiedBy: Denis Gradinarov; dateIdentified: 2026; **Event:** samplingProtocol: at light; verbatimEventDate: 28.vi.2019–29.vi.2019; habitat: xerothermic vegetation
- **Type status:** Other material. **Occurrence:** catalogNumber: BFUS-COL000861; recordedBy: Ognyan Sivilov & Boyan Zlatkov; individualCount: 1; sex: specimen; occurrenceID: 7936D41E-E981-58B0-A207-2D29D811136F; **Location:** country: Bulgaria; stateProvince: Blagoevgrad; municipality: Kresna; locality: Kresnenski Prolom Gorge, Sheitan Dere; verbatimElevation: 205 m; decimalLatitude: 41.760583; decimalLongitude: 23.154583; geodeticDatum: WGS84; **Identification:** identifiedBy: Denis Gradinarov; dateIdentified: 2026; **Event:** samplingProtocol: at light; verbatimEventDate: 01.vii.2019–02.vii.2019
- **Type status:** Other material. **Occurrence:** catalogNumber: BFUS-COL000862; recordedBy: Ognyan Sivilov & Boyan Zlatkov; individualCount: 1; sex: specimen; occurrenceID: AB11B86E-C0CF-58E3-A303-02512993D770; **Location:** country: Bulgaria; stateProvince: Blagoevgrad; municipality: Kresna; locality: Kresnenski Prolom Gorge, Sheitan Dere; verbatimElevation: 205 m; decimalLatitude: 41.760583; decimalLongitude: 23.154583; geodeticDatum: WGS84; **Identification:** identifiedBy: Denis Gradinarov; dateIdentified: 2026; **Event:** samplingProtocol: at light; verbatimEventDate: 01.vii.2019–02.vii.2019
- **Type status:** Other material. **Occurrence:** catalogNumber: BFUS-COL000863; recordedBy: Denis Gradinarov; individualCount: 1; sex: male; occurrenceID: B226966D-538B-5C67-A0CA-35669D37D565; **Location:** country: Bulgaria; stateProvince: Varna; municipality: Varna; locality: NE of Varna, University Botanical Garden; verbatimElevation: 59 m; decimalLatitude: 43.236967; decimalLongitude: 28.003917; geodeticDatum: WGS84; **Identification:** identifiedBy: Denis Gradinarov; dateIdentified: 2026; **Event:** samplingProtocol: at light; verbatimEventDate: 11.vii.2024; habitat: riverine forest

##### Distribution

Azerbaijan, Austria, Bulgaria, Czechia, France, Germany, Greece, Hungary, Italy, Portugal, Romania, Slovakia, Spain, South European Territory of Russia, Switzerland, Ukraine, Cyprus, Iran, Asian Türkiye, Algeria (Schuh 2020).

##### Notes

**Figure 13.**
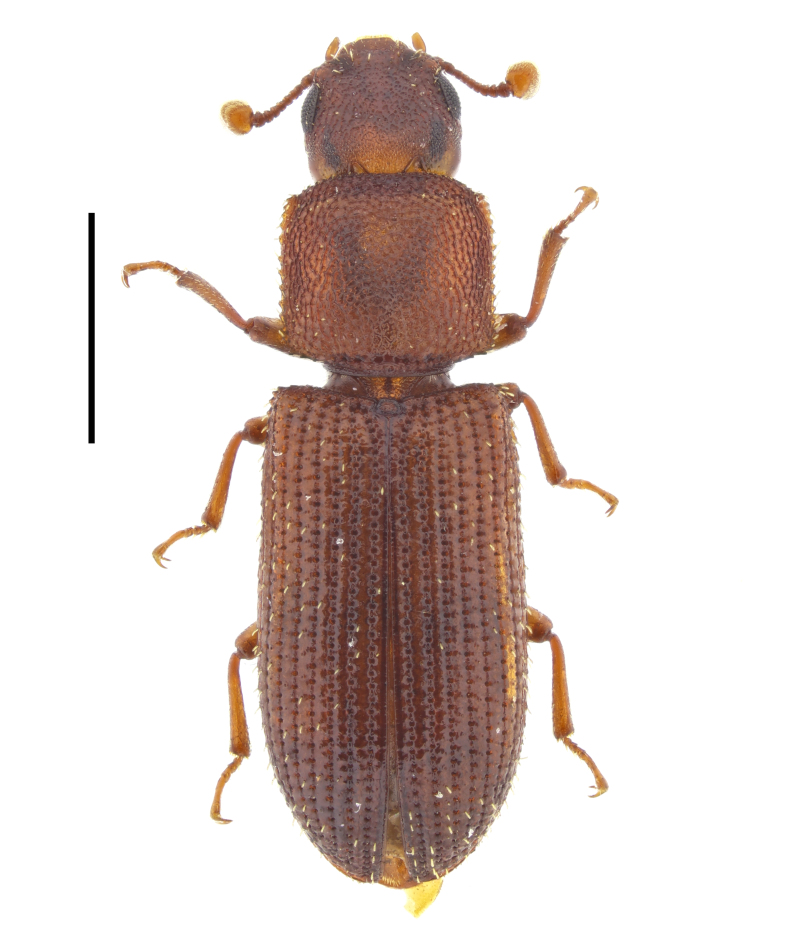
*Synchita mediolanensis* A. Villa & J. B. Villa, 1833 from Varna, Bulgaria (male, BFUS-COL000863). Scale bar: 1 mm.

In Bulgaria, this species has been previously recorded without exact localities (Ślipiński and Schuh 2008: 84, Schuh 2020: 75). Herein, we report *S. mediolanensis* from Varna (Fig. 13) and from several localities in south-western Bulgaria, confirming the presence of the species in the country.

#### Synchita separanda

(Reitter, 1882)

75B25C38-1836-59C2-87B1-4F5F86424760

##### Distribution

Armenia, Austria, Bosnia and Herzegovina, Bulgaria, Croatia, Czechia, Finland, France, Great Britain, Germany, Hungary, Italy, The Netherlands, Norway, Poland, Romania, Russia (South, Central and North European Territory), Slovakia, Spain, Sweden, Switzerland, Ukraine, Iran, Asian Türkiye (Schuh 2020); Belgium (Thomaes et al. 2025).

##### Notes

In Bulgaria, this species has recently been reported from southern Dobrudzha (Trigortsi Village) (Guéorguiev et al. 2026: 4).

#### Synchita undata

Guérin-Méneville, 1844

FD1596FB-92F8-5776-A1A5-109DD3F2CABA

##### Materials

- **Type status:** Other material. **Occurrence:** catalogNumber: BFUS-COL000864; recordedBy: Denis Gradinarov; individualCount: 1; sex: male; occurrenceID: DB2BF278-6A11-52F6-B052-490F0062AFB5; **Location:** country: Bulgaria; stateProvince: Sofia City; municipality: Stolichna; locality: Sofia City, Borisova Gradina Park; verbatimElevation: 579 m; decimalLatitude: 42.679133; decimalLongitude: 23.335667; geodeticDatum: WGS84; **Identification:** identifiedBy: Denis Gradinarov; dateIdentified: 2026; **Event:** samplingProtocol: under bark of linden stump; verbatimEventDate: 11.iv.2025; habitat: mixed forest
- **Type status:** Other material. **Occurrence:** catalogNumber: BFUS-COL000865; recordedBy: Denis Gradinarov; individualCount: 1; sex: male; occurrenceID: 338CDE1F-0F9B-57B8-BDC0-AC4A702F0E69; **Location:** country: Bulgaria; stateProvince: Sofia City; municipality: Stolichna; locality: Sofia City, Borisova Gradina Park; verbatimElevation: 579 m; decimalLatitude: 42.679133; decimalLongitude: 23.335667; geodeticDatum: WGS84; **Identification:** identifiedBy: Denis Gradinarov; dateIdentified: 2026; **Event:** samplingProtocol: under bark of linden stump; verbatimEventDate: 11.iv.2025; habitat: mixed forest
- **Type status:** Other material. **Occurrence:** catalogNumber: BFUS-COL000866; recordedBy: Denis Gradinarov; individualCount: 1; sex: male; occurrenceID: 5CCBF18D-B321-513A-B602-06ABC63CF2B3; **Location:** country: Bulgaria; stateProvince: Sofia City; municipality: Stolichna; locality: Sofia City, Borisova Gradina Park; verbatimElevation: 579 m; decimalLatitude: 42.679133; decimalLongitude: 23.335667; geodeticDatum: WGS84; **Identification:** identifiedBy: Denis Gradinarov; dateIdentified: 2026; **Event:** samplingProtocol: under bark of linden stump; verbatimEventDate: 11.iv.2025; habitat: mixed forest
- **Type status:** Other material. **Occurrence:** catalogNumber: BFUS-COL000867; recordedBy: Denis Gradinarov; individualCount: 1; sex: female; occurrenceID: BCAEA6DE-9433-5D0E-AE73-FA8D32F0F82B; **Location:** country: Bulgaria; stateProvince: Sofia City; municipality: Stolichna; locality: Sofia City, Borisova Gradina Park; verbatimElevation: 579 m; decimalLatitude: 42.679133; decimalLongitude: 23.335667; geodeticDatum: WGS84; **Identification:** identifiedBy: Denis Gradinarov; dateIdentified: 2026; **Event:** samplingProtocol: under bark of linden stump; verbatimEventDate: 11.iv.2025; habitat: mixed forest
- **Type status:** Other material. **Occurrence:** catalogNumber: BFUS-COL000868; recordedBy: Denis Gradinarov; individualCount: 1; sex: female; occurrenceID: 12BE0BE1-DE76-5DCC-A0F1-7B5DC48161FA; **Location:** country: Bulgaria; stateProvince: Sofia City; municipality: Stolichna; locality: Sofia City, Borisova Gradina Park; verbatimElevation: 579 m; decimalLatitude: 42.679133; decimalLongitude: 23.335667; geodeticDatum: WGS84; **Identification:** identifiedBy: Denis Gradinarov; dateIdentified: 2026; **Event:** samplingProtocol: under bark of linden stump; verbatimEventDate: 11.iv.2025; habitat: mixed forest
- **Type status:** Other material. **Occurrence:** catalogNumber: BFUS-COL000869; recordedBy: Denis Gradinarov; individualCount: 1; sex: female; occurrenceID: EE5DAA97-971E-5BA4-92E3-BA73B38E6CC7; **Location:** country: Bulgaria; stateProvince: Sofia City; municipality: Stolichna; locality: Sofia City, Borisova Gradina Park; verbatimElevation: 579 m; decimalLatitude: 42.679133; decimalLongitude: 23.335667; geodeticDatum: WGS84; **Identification:** identifiedBy: Denis Gradinarov; dateIdentified: 2026; **Event:** samplingProtocol: under bark of linden stump; verbatimEventDate: 11.iv.2025; habitat: mixed forest

##### Distribution

Austria, Bosnia and Herzegovina, Bulgaria, Czechia, France, Great Britain, Germany, Hungary, Italy, Romania, Portugal, Slovenia, South European Territory of Russia, Switzerland, Ukraine (Schuh 2020); Poland (Ruta 2020); Iran (Farashiani et al. 2022); Moldova (Bacal 2023); Asian Türkiye (Sürgüt and Varli 2023); Belgium (Thomaes et al. 2025).

##### Notes

**Figure 14.**
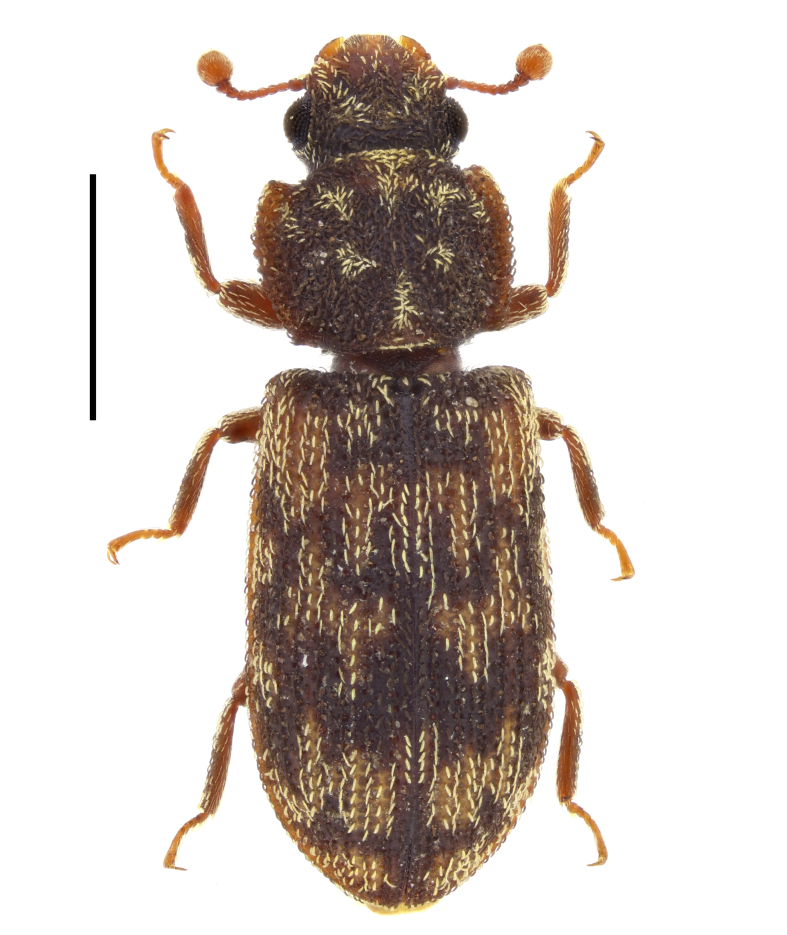
*Synchita undata* Guérin-Méneville, 1844 from Sofia City, Bulgaria (male, BFUS-COL000864). Scale bar: 1 mm.

In Bulgaria, this species has recently been reported from southern Dobrudzha (Trigortsi Village) and from Petrich (Guéorguiev et al. 2026: 4). Here, we report *S. undata* from the city park in Sofia (Fig. 14).

#### Synchita variegata

Hellwig, 1792

C90E75DD-54F7-5288-8C95-DBCEFABC5218

##### Materials

- **Type status:** Other material. **Occurrence:** catalogNumber: BFUS-COL000870; recordedBy: Ognyan Sivilov; individualCount: 1; sex: male; occurrenceID: 5E19F76A-F855-50F5-9978-247D31CC0A3B; **Location:** country: Bulgaria; stateProvince: Blagoevgrad; municipality: Sandanski; locality: Pirin Mountains, SE of Pirin Village; verbatimElevation: 892 m; decimalLatitude: 41.521697; decimalLongitude: 23.578828; geodeticDatum: WGS84; **Identification:** identifiedBy: Denis Gradinarov; dateIdentified: 2026; **Event:** samplingProtocol: pitfall traps; verbatimEventDate: 06.iv.2012–16.v.2012; habitat: beech forest
- **Type status:** Other material. **Occurrence:** catalogNumber: BFUS-COL000871; recordedBy: Ognyan Sivilov; individualCount: 1; sex: specimen; occurrenceID: 05E69F97-3B2E-503D-B3C8-DE67207C103D; **Location:** country: Bulgaria; stateProvince: Blagoevgrad; municipality: Sandanski; locality: Pirin Mountains, SE of Pirin Village; verbatimElevation: 892 m; decimalLatitude: 41.521697; decimalLongitude: 23.578828; geodeticDatum: WGS84; **Identification:** identifiedBy: Denis Gradinarov; dateIdentified: 2026; **Event:** samplingProtocol: pitfall traps; verbatimEventDate: 06.iv.2012–16.v.2012; habitat: beech forest
- **Type status:** Other material. **Occurrence:** catalogNumber: BFUS-COL000872; recordedBy: Ognyan Sivilov; individualCount: 1; sex: male; occurrenceID: 68E5D325-3957-5287-B340-EE9C38C4FA1B; **Location:** country: Bulgaria; stateProvince: Blagoevgrad; municipality: Sandanski; locality: Pirin Mountains, SE of Pirin Village; verbatimElevation: 892 m; decimalLatitude: 41.521697; decimalLongitude: 23.578828; geodeticDatum: WGS84; **Identification:** identifiedBy: Denis Gradinarov; dateIdentified: 2026; **Event:** samplingProtocol: pitfall traps; verbatimEventDate: 16.v.2012–17.vi.2012; habitat: beech forest
- **Type status:** Other material. **Occurrence:** catalogNumber: BFUS-COL000873; recordedBy: Ognyan Sivilov; individualCount: 1; sex: female; occurrenceID: B70A0769-8AFA-5BF5-A660-4EFC4FEDEC5F; **Location:** country: Bulgaria; stateProvince: Blagoevgrad; municipality: Sandanski; locality: Pirin Mountains, SE of Pirin Village; verbatimElevation: 892 m; decimalLatitude: 41.521697; decimalLongitude: 23.578828; geodeticDatum: WGS84; **Identification:** identifiedBy: Denis Gradinarov; dateIdentified: 2026; **Event:** samplingProtocol: pitfall traps; verbatimEventDate: 16.v.2012–17.vi.2012; habitat: beech forest
- **Type status:** Other material. **Occurrence:** catalogNumber: BFUS-COL000874; recordedBy: Ognyan Sivilov; individualCount: 1; sex: female; occurrenceID: BB5A32F5-A248-5767-8698-68933C728019; **Location:** country: Bulgaria; stateProvince: Blagoevgrad; municipality: Sandanski; locality: Pirin Mountains, SE of Pirin Village; verbatimElevation: 892 m; decimalLatitude: 41.521697; decimalLongitude: 23.578828; geodeticDatum: WGS84; **Identification:** identifiedBy: Denis Gradinarov; dateIdentified: 2026; **Event:** samplingProtocol: pitfall traps; verbatimEventDate: 16.v.2012–17.vi.2012; habitat: beech forest
- **Type status:** Other material. **Occurrence:** catalogNumber: BFUS-COL000875; recordedBy: Ognyan Sivilov; individualCount: 1; sex: female; occurrenceID: DBE03ADB-95C6-5EA9-AC6C-5062FEAB8667; **Location:** country: Bulgaria; stateProvince: Blagoevgrad; municipality: Sandanski; locality: Pirin Mountains, SE of Pirin Village; verbatimElevation: 892 m; decimalLatitude: 41.521697; decimalLongitude: 23.578828; geodeticDatum: WGS84; **Identification:** identifiedBy: Denis Gradinarov; dateIdentified: 2026; **Event:** samplingProtocol: pitfall traps; verbatimEventDate: 10.ix.2012–18.x.2012; habitat: beech forest
- **Type status:** Other material. **Occurrence:** catalogNumber: BFUS-COL000876; recordedBy: Ognyan Sivilov; individualCount: 1; sex: male; occurrenceID: 4804D9FC-87D2-5E6B-A3C6-CF7580665554; **Location:** country: Bulgaria; stateProvince: Blagoevgrad; municipality: Sandanski; locality: Pirin Mountains, SE of Pirin Village; verbatimElevation: 896 m; decimalLatitude: 41.521558; decimalLongitude: 23.578914; geodeticDatum: WGS84; **Identification:** identifiedBy: Denis Gradinarov; dateIdentified: 2026; **Event:** samplingProtocol: pitfall traps; verbatimEventDate: 10.ix.2012–18.x.2012; habitat: beech forest
- **Type status:** Other material. **Occurrence:** catalogNumber: BFUS-COL000877; recordedBy: Ognyan Sivilov; individualCount: 1; sex: male; occurrenceID: 2593CB5D-CCAF-5BE0-A6EF-20DC7EE3E312; **Location:** country: Bulgaria; stateProvince: Blagoevgrad; municipality: Gotse Delchev; locality: Pirin Mountains, NW of Popovi Livadi Village; verbatimElevation: 1562 m; decimalLatitude: 41.556036; decimalLongitude: 23.629097; geodeticDatum: WGS84; **Identification:** identifiedBy: Denis Gradinarov; dateIdentified: 2026; **Event:** samplingProtocol: pitfall traps; verbatimEventDate: 18.vi.2012–20.vii.2012; habitat: beech forest

##### Distribution

Austria, Belgium, Bosnia and Herzegovina, Bulgaria, Croatia, Czechia, France, Great Britain, Germany, Greece, Hungary, Italy, Liechtenstein, The Netherlands, Portugal, Romania, Russia, Slovenia, Spain, Sweden, Switzerland, Ukraine, Serbia and Montenegro, European and Asian Türkiye, Algeria, Nearctic Region (introduced) (Schuh 2020); Poland (Ruta 2020); Belgium (Thomaes et al. 2025).

##### Notes

**Figure 15.**
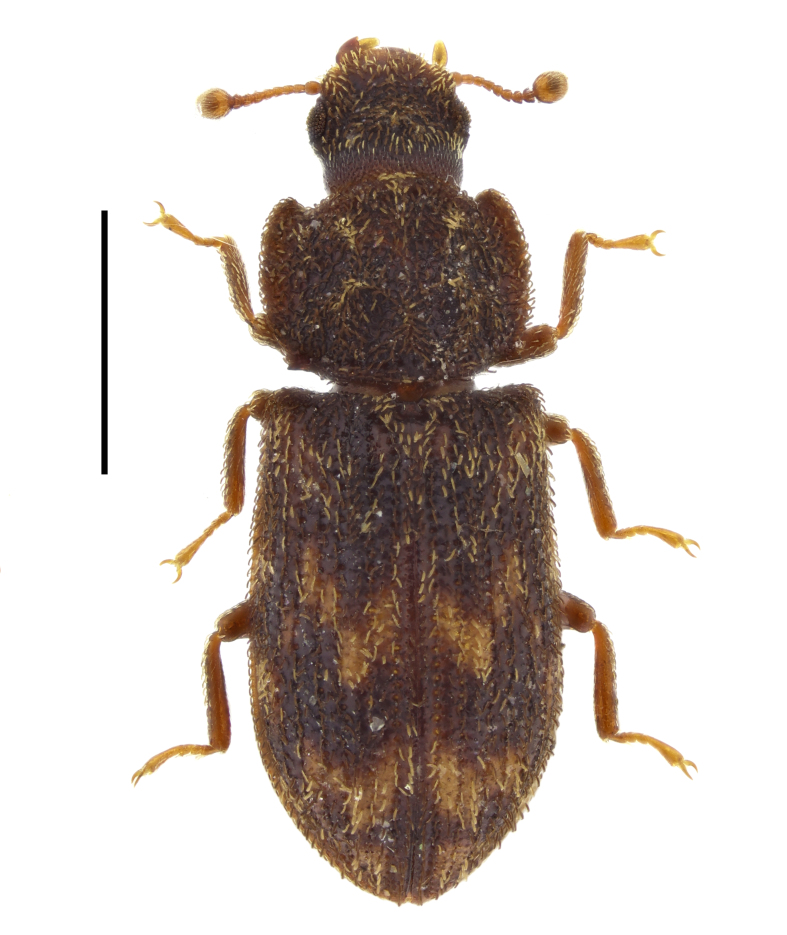
*Synchita variegata* Hellwig, 1792 from Pirin Mountains, Bulgaria (male, BFUS-COL000872). Scale bar: 1 mm.

In Bulgaria, *S. variegata* is known from Varshets (Western Stara Planina Mountains) (Schuh 1998: 318). We found this species in beech forests of Pirin Mountains (Fig. 15).

## Discussion

Twenty-one species of the family Zopheridae are listed for Bulgaria. One of these species – *Colydium noblecourti* is newly reported for the country in the present study. For four species – *Aulonium trisulcum*, *Aulonium ruficorne*, *Langelandia anophthalma* and *Synchita mediolanensis*, we provide exact localities and thus confirm their occurrence in Bulgaria. *Orthocerus clavicornis* is reported for the second time from the country after nearly 60 years of lack of data. For the species *Rhopalocerus rondanii* and *Synchita humeralis*, no published exact localities in Bulgaria are available and further studies on their distribution in the country are desirable.

## Supplementary Material

XML Treatment for Coleoptera

XML Treatment for Tenebrionoidea

XML Treatment for Zopheridae

XML Treatment for Zopherinae

XML Treatment for Pycnomerini

XML Treatment for Pycnomerus

XML Treatment for Pycnomerus sulcicollis

XML Treatment for Pycnomerus terebrans

XML Treatment for Colydiinae

XML Treatment for Colydiini

XML Treatment for Aulonium

XML Treatment for Aulonium ruficorne

XML Treatment for Aulonium trisulcum

XML Treatment for Colydium

XML Treatment for Colydium elongatum

XML Treatment for Colydium filiforme

XML Treatment for Colydium noblecourti

XML Treatment for Orthocerini

XML Treatment for Orthocerus

XML Treatment for Orthocerus clavicornis

XML Treatment for Rhopalocerini

XML Treatment for Rhopalocerus

XML Treatment for Rhopalocerus rondanii

XML Treatment for Synchitini

XML Treatment for Bitoma

XML Treatment for Bitoma crenata

XML Treatment for Colobicus

XML Treatment for Colobicus hirtus

XML Treatment for Endophloeus

XML Treatment for Endophloeus markovichianus

XML Treatment for Endophloeus squarrosus

XML Treatment for Langelandia

XML Treatment for Langelandia

XML Treatment for Langelandia (Langelandia) anophthalma

XML Treatment for Nosodomodes

XML Treatment for Nosodomodes diabolicus

XML Treatment for Nosodomodes tuberculatus

XML Treatment for Synchita

XML Treatment for Synchita humeralis

XML Treatment for Synchita mediolanensis

XML Treatment for Synchita separanda

XML Treatment for Synchita undata

XML Treatment for Synchita variegata
